# Experiential therapies including Chairwork: a systematic review of randomized controlled trials

**DOI:** 10.3389/fpsyg.2025.1692630

**Published:** 2026-01-23

**Authors:** Lenka Ottingerová, Júlia Halamová, Dagmar Szitás

**Affiliations:** Faculty of Social and Economic Sciences, Institute of Applied Psychology, Comenius University in Bratislava, Bratislava, Slovakia

**Keywords:** Chairwork, empty chair, intervention, psychotherapy, systematic review, two chair, unfinished business

## Abstract

Chairwork refers to a set of experiential psychotherapeutic interventions in which the physical positioning of the chairs facilitates internal and interpersonal dialogue. This study is an attempt to rectify the lack of a systematic review of the research on the effectiveness of Chairwork by offering a systematic review of randomized controlled trials (RCTs) that assess the effectiveness of Chairwork-based interventions used in various psychotherapeutic approaches for the treatment of psychological disorders, difficulties, and mental health conditions. It considers various psychotherapy modalities and formats, including individual, couple, group, and family therapies. We followed the updated criteria of the Preferred Reporting Items for Systematic Review. Our systematic review examines the relevant literature from two scientometric databases: Web of Science and Scopus published up to August 30, 2024. An independent assessment of the risk of bias in the studies was performed using the Cochrane Risk of Bias 2.0 along with a certainty assessment. A total of 22 RCTs were included in the final analysis, providing robust empirical support for Chairwork’s efficacy across several clinical domains, including depression, childhood trauma, unfinished business, obsessive-compulsive disorder (OCD), post-traumatic stress disorder (PTSD), social anxiety, and eating disorders. The results demonstrated efficacy across different therapeutic approaches, with Chairwork utilized both as a core experiential component within broader frameworks (e.g., EFT) and as a stand-alone intervention. Effect sizes (Cohen’s d) varied depending on the outcome measure, ranging from small (*d* = 0.20) to large (*d* = 1.73). Our findings show that Chairwork seems to be a promising psychotherapeutic intervention for individuals, groups, and families. We provide a detailed analysis of these findings. We did not find any relevant studies relating to couple therapy.

## Introduction

Psychotherapeutic interventions play a crucial role in addressing various mental health problems. They offer an extensive, evidence-based approach to treatment and emphasize the importance of individual agency and empowerment in the recovery process (Cook et al., 2017). Among these diverse approaches, Experiential Therapies represent a psychotherapeutic approach that emphasizes emotional activation, the symbolization of internal experience, and the transformation of maladaptive emotional states through direct in-session processing (Elliott et al., 2021; Greenberg et al., 1989).

Experiential therapies share the premise of facilitating emotional transformation and psychological change by activating, deepening, and reorganizing clients’ core emotional experiences in session (Greenberg, 2004). This framework emphasizes working through difficulties by activating emotion in session, symbolizing emotional experience, and engaging clients in processes that foster this transformation. Through experiential processing, clients are supported to articulate previously unexpressed feelings, confront internal conflicts, and develop adaptive emotional responses that promote psychological integration and behavioral change (Auszra et al., 2013; Pascual-Leone and Greenberg, 2007). Proposed mechanisms of change, which provide the conceptual grounding for these approaches, include increased emotional awareness, improved emotion regulation, corrective emotional experiences, and the restructuring of maladaptive emotion schemes. This foundational framework sets the stage for specific techniques that operationalize these goals.

Within this framework, Chairwork is an experiential psychotherapy technique where role-playing is the central component, enabling clients to directly engage with and examine distinct aspects of their psyche or relationships with significant others (Kellogg, 2004). Two enactments are used: Two Chair (the self-critical split) and Empty Chair (unfinished business) to support clients when expressing and deepening their unrecognized emotions and acknowledging different parts of the self. Although two-chair and empty-chair enactments are among the most widely researched forms, Chairwork includes a broader range of dialogical configurations, emotional processing tasks, and experiential structures (Kellogg, 2004). By giving voice to different parts of the self or relational dynamics, these techniques facilitate emotional deepening and self-exploration, ultimately leading clients to resolve their inner conflicts and unmet needs and achieve the desired emotional transformation (Elliott and Greenberg, 1997; Greenberg, 2004). This demonstration establishes how Chairwork functions as a central method through which the aims of the broader experiential approach are achieved.

As Chairwork continues to gain recognition within the field of psychotherapy, research efforts have focused on verifying its effectiveness across different populations and mental health problems. It has been shown to be effective in treating a variety of psychological conditions, such as psychosis (Chadwick, 2003) and borderline personality disorder (van Maarschalkerweerd et al., 2021). Chairwork dialogue has helped individuals address various psychological difficulties, including anger management (e.g., Conoley et al., 1983; Narkiss-Guez et al., 2015), interpersonal conflict resolution (Greenberg and Dompierre, 1981; Greenberg and Higgins, 1980), and trauma recovery (Paivio et al., 2010). Another effective method involves clients dramatizing situations to challenge negative thoughts and beliefs (e.g., de Oliveira et al., 2012; Delavechia et al., 2016), or self-criticism (Falconer et al., 2014; Shahar et al., 2012). Furthermore, the versatility of Chairwork in addressing a wide range of issues means it has potential as a valuable tool in the therapeutic toolkit.

Recent empirical evidence further shows that Chairwork, when used as a stand-alone intervention (often in single-session or dismantling trials), reliably increases depth of experiencing and facilitates emotional transformation (Pascual-Leone and Baher, 2023; Baher, 2023). These findings underscore the role of chair-based enactments as a core method through which experiential therapies achieve their therapeutic outcomes.

## Background

Chairwork refers to a set of experiential therapeutic interventions in which the physical positioning of the chairs facilitates internal and interpersonal dialogue (Pugh, 2017). This technique has evolved out of various therapeutic approaches. Its roots can be traced back to the early 20th century, and the pioneering work of Jacob Moreno, the founder of psychodrama (Moreno, 1948; Fox, 1987). Moreno (1948) recognized the therapeutic potential of using physical space and movement to facilitate emotional expression and interpersonal exploration. Using these techniques, clients can externalize and address diverse aspects of their inner experience, including emotional conflicts, schema-level beliefs, and fragmented self-representations. Furthermore, these dialogues have multiple therapeutic applications, including work with internal parts, conflicting needs, significant others, imagined scenarios, and unresolved experiences (Greenberg, 2002; Kellogg, 2004).

The Chairwork process consists of several stages. It commences with the identification of relevant issues or emotions, which are then externalized to the empty chairs. The main components of this process are emotional expression, dialogue, perspective-taking, and integrating insights (Greenberg and Watson, 2006). Proceeding through these stages, individuals ultimately seek to transform their emotions, resolve the problem, and achieve personal growth (Greenberg, 2004). Chairwork encompasses a diverse range of experiential techniques in which chairs are used to facilitate therapeutic processes. Two prominent forms of Chairwork are the Empty Chair Technique and the Two Chair Technique.

### Empty chair technique

Unresolved feelings toward significant others, characterized by persistent negative emotions, such as the hurt and resentment stemming from past experiences of neglect, abandonment, or abuse, perpetuate the longing associated with unmet interpersonal needs in these relationships. This unprocessed emotional state exerts an influence on present-day relationships (Elliott et al., 2004). In this technique, two chairs are placed facing each other, with the client representing significant others. Empty Chairwork for unfinished business facilitates the emotional processing of these unresolved negative feelings by enabling clients to access them and communicate them to an imagined significant other (Goldman and Greenberg, 2015). This guided process of re-experiencing and addressing previously inhibited primary emotions, such as sadness and anger, empowers clients so they can legitimize their entitlement to unmet interpersonal needs and assert themselves when confronted with the imagined other (Greenberg and Watson, 2006). By switching between chairs, clients can engage in dialogues that promote self-awareness, emotional expression, and the resolution of conflicts with their significant other (Greenberg et al., 1996). Depending on the nature of the unfinished business, the resolution of the emotional processing difficulty may involve a shift in the client’s perspective on the significant other, either through enhanced understanding leading to forgiveness or holding the other accountable for past injury (Elliott et al., 2004; Greenberg and Watson, 2006).

### Two chair technique

The Two Chair Technique involves the use of two chairs to represent conflicting aspects, through a structured interaction where the individual alternately embodies the perspectives of their inner critic and their experiential self (Elliott et al., 2004). The client then moves to the chair representing their experiential self, where they are encouraged to explore and articulate the emotional reactions evoked by the self-critical attacks, which often involve feelings of powerlessness and despair. The individual alternates between the chairs, adopting different perspectives and engaging in a dialogue that fosters awareness and the integration of conflicting emotions or beliefs (Greenberg and Watson, 2006). The resolution of this emotional processing often involves asserting the experiencing self’s needs and the subsequent transformation of the previously harsh inner critic into a compassionate stance. This emotional transformation involves a shift on both sides, followed by a negotiation between the two parts to achieve integration, which aligns the experiencing self’s needs with the inner critic’s values and standards (Elliott et al., 2004; Greenberg and Watson, 2006).

### Differences in Chairwork across therapy orientations

While psychotherapeutic interventions may share certain core principles, the various modalities may have a distinct vision of the therapeutic process, the desired outcomes, and the role of the therapist. These unique perspectives can shape the specific techniques, goals, and relational dynamics within the therapeutic encounter (Kellogg, 2004). Chairwork has been integrated into various therapeutic approaches, each with its unique emphasis and application.

Gestalt therapy emphasizes the importance of integration and wholeness, focusing on the present moment and the individual’s awareness of their thoughts, feelings, and actions (Perls, 1969). In Chairwork, this theoretical foundation translates into a tangible representation of the person’s internal conflicts, allowing dynamic dialogue with various aspects of their personality, memories, or significant individuals (Greenberg et al., 1996). Perls (1969) further developed the use of chairs in therapy by encouraging clients to confront and explore internal conflicts and unresolved emotional experiences. Clients explore their inner conflicts by imagining themselves in a scenario with two opposing viewpoints. By actively engaging with each perspective (often by switching back and forth between them), the client aims to gain new insights, feelings, or a broader understanding of their struggles. This process can ultimately lead to a resolution of the conflict or a shift in emotions (Greenberg et al., 1996; Greenberg and Watson, 2006; Perls, 1973).

Voice therapy is a form of experiential psychotherapy that employs Chairwork to explore and integrate multiple aspects of the self. This approach draws on principles from Gestalt therapy, voice dialogue, and the paradoxical theory of change (Perls, 1969; Stone and Stone, 1989; Beisser, 1970). The therapist facilitates the process by guiding the patient through the Chairwork. Initially, the patient sits in one chair, representing a healthy self. They then move to another chair to embody and express their suffering. The therapist listens attentively and may use techniques such as repetition, existential questions, and voice modulation to deepen the emotional experience. The goal is not to eliminate the pain but to allow its full expression. Finally, the patient returns to the “center chair” to reflect on the experience with the therapist (Kellogg and Garcia Torres, 2021). By contrast, Voice Therapy is a powerful technique that quickly taps into the client’s core negative beliefs. It involves a process of identifying and eliciting the negative thought patterns (“critical inner voices”) driving the person’s maladaptive behavior (Firestone, 1987). The therapeutic power of voice therapy lies in its ability to confront internal conflicts. By creating a safe space for dialogue with these inner voices, clients can explore the origins of self-destructive patterns and negative self-beliefs (often stemming from internalized parental criticism). This process promotes emotional catharsis (release), leading to greater self-acceptance and a more compassionate internal narrative (Firestone, 2018).

Over time, Chairwork has successfully integrated cognitive-behavioral principles, enriching its scope and effectiveness. This integration enables individuals to explore their emotions and experiences and challenge and reframe maladaptive beliefs and thought patterns (Leahy, 2003). By engaging in constructive dialogues with different chair representations, individuals gain a more comprehensive understanding of their cognitive-emotional conflicts, paving the way for therapeutic transformation (Pugh and Bell, 2020). When utilizing Chairwork to restructure cognition, a two-chair format is commonly employed. One chair represents the supporting evidence for a thought or belief, while the second chair embodies disconfirmatory evidence. The specific sequence of this two-chair cognitive restructuring process can vary depending on the client’s level of conviction and emotional investment in the belief (Pos and Greenberg, 2012). Furthermore, some experts emphasize the potential value of incorporating a third “empathic” chair, which cultivates a compassionate and care-focused perspective. This additional chair may be particularly beneficial if the client tends to invalidate their emotional experiences or when self-directed care appears to be lacking (Beck et al., 1979). It is important to note that two-chair cognitive restructuring exercises often require repeated practice to facilitate sufficient socialization and achieve the desired therapeutic effects (Ociskova et al., 2022).

Schema therapy leverages Chairwork to address internal conflicts through dialogues between distinct schema modes. These modes, unlike typical internal voices, are not simply metaphorical representations, but rather well-defined patterns encompassing emotions, cognitions, behaviors, and physiological responses (Young et al., 2003). Schema modes can be healthy (e.g., Healthy Adult) or unhealthy (e.g., Vulnerable Child, Angry Child, Critical Parent). By embodying these modes in chairs, clients engage in a structured and focused exploration of their internal dynamics. The therapist and client collaboratively identify a target issue, and the therapist arranges chairs to represent characters or aspects of the client’s experience. These can be from the past, present, or future, or they can be internal voices such as the critical self, coping mechanisms, or even a healthy adult perspective. Through dialogues enacted in these chairs, clients delve deeper into their internal landscape, working toward resolving distress (Ociskova et al., 2022).

Emotion-Focused Therapy (EFT) is a humanistic psychotherapeutic approach that prioritizes the significance of emotional experiences and their expression within the therapeutic context (Greenberg, 2004). The foundation of EFT’s therapeutic efficacy is the emotional support within the therapy, which is guided by person-centered, emotion-processing principles (Goldman, 2017; Greenberg, 2015; Pascual-Leone and Greenberg, 2007): (1) Identifying primary maladaptive emotions obscured by secondary, symptomatic emotions. Once these primary emotions are accessed, adaptive emotional responses can be utilized to transform the maladaptive emotional schemes that generate chronic distress and sustain problematic behavioral patterns. (2) Facilitating Emotional Expression: Chairwork encourages clients to externalize and articulate their emotions, increasing their emotional clarity and intensity. By speaking from different perspectives, clients can explore and express feelings that may have previously remained unvoiced. Through Chairwork, clients can better understand the various aspects of their emotional experience. This heightened self-awareness is essential for recognizing patterns of behavior and emotional responses. (3) Accessing and Transforming Emotions: Chairwork enables clients to engage deeply with their emotions, facilitating the identification and transformation of maladaptive emotional responses. This is crucial for resolving emotional conflicts and promoting emotional regulation. By entering into dialogue with different parts of themselves or with imagined others, clients can develop a more compassionate and empathetic view of their experiences, leading to greater self-acceptance and healing (Goldman, 2017; Greenberg, 2015; Pascual-Leone and Greenberg, 2007).

To address and systematize the heterogeneous application of Chairwork across various theoretical models, Kellogg and Garcia Torres (2021) proposed the Four Dialogues framework. This model provides a unifying structure for understanding the technique by categorizing its usage into four core applications: Giving Voice, Internal Dialogues, Telling the Story, and Relationships and Encounters. This systematization allows clinicians to move beyond a single theoretical orientation (e.g., Gestalt or EFT) and apply Chairwork purposefully for healing and transformation based on the client’s current therapeutic need (Kellogg and Garcia Torres, 2021).

### The research aim

Although recent meta-analytic evidence demonstrates that Chairwork is effective when delivered as a stand-alone intervention (Pascual-Leone and Baher, 2023; Baher, 2023), clinical practice typically integrates chair-based methods within broader treatment packages (e.g., EFT, schema therapy, or trial-based cognitive therapy). This raises a crucial empirical question: Do therapies that include Chairwork as one component continue to demonstrate superior outcomes, or are the effects diluted when embedded in multimodal interventions?

This systematic review addresses this distinction by synthesizing the evidence on the effectiveness of broader treatment protocols that explicitly include Chairwork, as assessed exclusively through randomized controlled trial (RCT) designs, thereby providing a clearer picture of its overall clinical utility across diverse populations and disorders.

## Materials and methods

### Methods

To ensure transparency and accuracy in reporting the methodology and results of our systematic review, we followed the updated criteria of the Preferred Reporting Items for Systematic Review (PRISMA; Page et al., 2021). Adherence to PRISMA guidelines minimizes bias and enhances the reproducibility of our study. Additionally, the RCTs were assessed for risk of bias using the Cochrane Risk of Bias 2.0 tool (Sterne et al., 2019), which evaluates the risk of bias across five domains: (1) randomization process, (2) deviations from intended interventions, (3) missing outcome data, (4) measurement of the outcome, and (5) selection of the reported results.

### Source of information

Our systematic review involved the examination of relevant literature from two scientometric databases: Web of Science and Scopus. The search strategy was keyword-based to ensure a comprehensive and targeted search for studies about Chairwork in psychotherapy. The scope of our review encompassed all the literature in English published up to August 30, 2024.

### Search strategy

Within the two databases, Web of Science and Scopus, we scrutinized records, including titles, abstracts, and keywords, where available. To ensure all relevant studies were included in the search, the search strategy was structured into three conceptual segments. The first segment targeted studies that examined Chairwork and related experiential dialogue-based techniques, using a broad set of synonyms such as “Chairwork,” “chair-work,” “empty chair,” “two-chair,” “unfinished business,” “hot seat,” “imaginal dialogue,” “self-critical chair,” “self-interruptive chair,” “self-soothing chair,” “compassionate chair,” “multiple selves,” and “voice dialogue.” The second segment focused on terms related to psychotherapeutic contexts, including “psychotherapy,” “therapy,” “counseling,” “counselling,” and “intervention.” This ensured that only studies using these methods within a therapeutic framework were identified. The third segment targeted treatment outcomes, combining terms such as “outcome,” “change,” “effect,” “efficacy,” and “effectiveness.” These terms captured studies evaluating the impact of experiential or chair-based interventions. To ensure full replicability, the complete search syntax used for each database is reported in Appendix A (see Supplementary material). The search or specification of matching syntagms within each database was performed in August 2024 by the first (LO) and third (DS) authors.

### Eligibility criteria

Articles were screened to ensure that the studies: (a) were conducted in the context of individual or couple or group or family psychotherapy or counseling session(s); (b) where Chairwork was facilitated (dialogue between different parts of the self or representation of others); (c) experiential treatments that include Chairwork components and (d) compared the quantitative outcomes of Chairwork with alternative interventions (i.e., between-group) or within the course of therapy (i.e., pre–post-treatment, multiple baseline). Additional criteria were the availability of the full study and English language.

### Selection process

All the authors contributed to defining the eligibility criteria for this systematic review. The first author conducted the initial database searches to identify potentially relevant studies. The second author then ensured that the established selection criteria were followed accurately during the study screening process. The search results were compiled in a single Microsoft Excel spreadsheet for further organization and analysis. The first author then reviewed the compiled list and removed any duplicate studies identified during the different database searches. Finally, the first and third authors independently reviewed the final list of studies. Where there was disagreement among the raters regarding the inclusion or exclusion of certain studies, the authors collaboratively discussed and resolved these issues until a consensus was reached on the final set of studies to be included in the review.

### Data collection

For each article included, the following information was obtained: general characteristics (authors, year of publication), type of study design (e.g., RCT, cross-sectional); type of technique or intervention; length of intervention; sample size(s) of subjects, comparative groups; assessment tools used in the study; outcome measures and lastly key findings based on the focus of our systematic review. See Table 1 for a complete list of the studies in alphabetical order and structured summaries. A separate Appendix C (see Supplementary material) containing the measurement tools used in the selected studies is provided.

**Table 1:** Final summary of selected studies.

Reference	Study type	Type of intervention	Length of intervention	Sample	Ratio in group	Measured construct
Ansar et al. (2022)	RCT	EFST: two-chair interventions Psychoeducational group (PED)	12-week (20 h)	236 parents	EFST (120) PED (116)	Mental health (parenting program)
Arimitsu (2016)	RCT (pilot study)	Enhancing Self-Compassion Program (ESP)—Three chair	7 weeks (1.5 h)	40 students	ESP (20) CG (20)	Self-compassion (group format)
Clarke (1981)	RCT	Two-Chair (TCH) Cognitive Problem-Solving (CPS)	6–12 weeks	48 adults	TCH (16) CPS (16) CG (16)	Carrier Decision Making
Diamond et al. (2016)	RCT	Attachment-Based Family Therapy (ABFT) Emotion-Focused Therapy (EFT)	10–16 weeks	32 adults	ABFT (16) EFT (16)	Unresolved anger (Family therapy)
Duran et al. (2020)	RCT	Trial-based cognitive therapy (TBCT) Prolonged exposure therapy (PE)	3–13 sessions (60 min)	95 patients	PE (51) TBCT (44)	Post-traumatic stress disorder (PTSD)
Ellison et al. (2009)	RCT	Client-centered therapy (CC) Emotion-focused therapy (EFT)	16–20 session	43 adults	CC (21) EFT (21)	Depression
Glisenti et al. (2021)	RCT (pilot study)	Emotion-focused therapy (EFT)	12 sessions (60 min)	21 clients	EFT (10) CG (11)	Eating disorder
Goldman et al. (2006)	RCT	Client-centered therapy (CC) Emotion-focused therapy (EFT)	16–20 sessions (50 min) Two sessions, 20-min segments	38 clients	CC (19) EFT (19)	Depression
Greenberg et al. (2008)	RCT	Empty Chair (ECH) Psychoeducational group (PED)	ECH–12 weeks (60 min)	46 clients	ECH (23) PED (23)	Unfinished business (emotional injuries)
Greenberg and Watson (1998)	RCT	Process-experiential psychotherapy (PE) Client-centered psychotherapy (CC)	16–20 sessions once a week	34 adults	PE (17) CC (17)	Depression
Hagl et al. (2014)	Controlled Trial	Empty Chair—dialogical group (EC) Supportive group (SG)	7-week group sessions (2-h sessions)	119 women	SG (59) ECHG (60)	Unfinished business (trauma loss)
Chagigiorgis (2009)	RCT	Empty chair in Imaginal Confrontation (IC) Empathic Exploration (EE)	16–20 weeks (60 min)	47 adults	IC (21) EE (26)	Childhood trauma
de Oliveira et al. (2012)	Two-arm RCT comparing	Trial-based thought record (TBTR) Conventional cognitive therapy (CCT)	2 one-hour individual sessions over 10 weeks, followed by sessions every 2 weeks for the last 4 weeks, spanning a total duration of 4 months.	36 patients	TBTR (17) CCT (19)	Social anxiety (core belief)
Paivio et al. (2010)	RCT (mixed-methods design)	Imaginal Confrontation (IC) Empathic Exploration (EE)	16–20 weekly (60 min)	45 adults	IC (20) EE (25)	Childhood trauma
Paivio and Greenberg (1995)	RCT	Empty Chair (ECH) psychoeducational group (PED)	ECH—12 weeks (50-min session) PED—two groups 3 sessions (2-h)	34 adults	ECH (17) PED (17)	Unfinished business (emotional injuries)
Parker (2007)	RCT	Cognitive Behavioral Therapy (CBT) Cognitive-Behavioral-Affective Therapy (CBAT)	4 (2-h sessions)	20 adults	CBT (10) CBAT (10)	Unfinished business (maladaptive anger)
Ralston (2006)	RCT (mixed-methods design)	EFTT: Imaginal confrontation (IC) Evocative empathy (EE)	16–20 weekly (60 min)	30 adults	IC (15) EE (15)	Childhood trauma
Rodrigues et al. (2023)	RCT	Trial-based cognitive therapy (TBCT) Exposure and response prevention (ERP)	12 sessions (60 min)	26 patients	TBCT (12) ERP (14)	Obsessive-compulsive disorder (OCD)
Souliere (1995)	RCT	Empty chair Cognitive restructuring	2 interventions for 2 weeks	50 adults	ECH (5) CR (5)	Unfinished business (lingering anger)
Stefan et al. (2023)	RCT	CST-based intervention	One-session intervention	112 adults	Ex. G (56) CG (56)	Social anxiety (online format)
Trachsel et al. (2012)	RCT	Two-chair approach (TCA) The decision-cube technique (DCT)	2 weeks	50 adults	TCA (25) DTC (25)	Partnership ambivalence
Watson et al. (2003)	RCT	Process-experimental therapy (PET) Cognitive-behavioral therapy (CBT)	16 weeks (60 min)	66 adults	PET (33) CBT (33)	Depression

### Risk of bias and certainty assessment

The reviewers independently assessed the risk of bias in the studies using the Cochrane Risk of Bias 2.0 (ROB-2; Sterne et al., 2019) tool. This tool evaluates the risk of bias across five domains: randomization process, deviations from intended interventions, missing outcome data, measurement of outcome, and selection of reported results. The criteria for reaching the overall judgments for the studies were applied following the guidelines for the ROB-2 tool (Sterne et al., 2019).

## Results

### Selection and description of eligible studies

**Figure 1:**
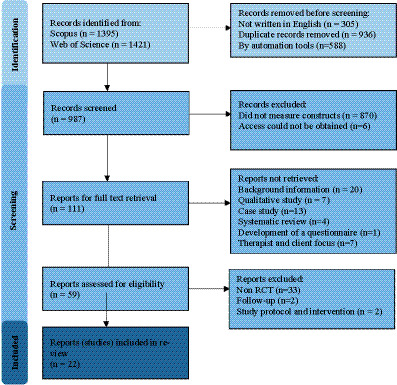
PRISMA 2020 flow diagram of selected reports.

Out of the initial pool of 2,816 articles, the preliminary exclusion round eliminated studies in a language other than English (*n* = 305). We then screened for duplicates, resulting in the removal of 936 redundant articles, see Figure 1 for a flowchart of the studies included. Ineligibility by automation tools was excluded based on the following categories: patent (*n* = 179), case report (*n* = 44), book (*n* = 5), book chapter (*n* = 31), data set (*n* = 7), editorial material (*n* = 22), conference paper (*n* = 37), and review article (*n* = 263).

A rigorous assessment of keyword interpretation led to the exclusion of 255 studies that contained the keyword(s) in the title but in a different context, such as the medical term “unfinished business” indicating persistent questions and areas of controversy in medical studies (Wilkie, 2014) or political studies (Kasara and Mares, 2017). Additionally, 615 studies with keywords with different contextual meanings, specifically related to voice therapy, e.g., voice patients’ recovery but not Chairwork (Kleemola et al., 2011) were removed. Moreover, six studies for which access could not be obtained were excluded (Johnson, 1976; McMain, 1995; O’grady, 1986; O’shea, 1982; Tsvieli and Diamond, 2018; Zichor, 2010).

Subsequent exclusions were made based on study type and content. The review excluded studies that primarily offered descriptive or theoretical overviews of therapeutic approaches without presenting original empirical data or detailed methodological evaluations. This includes papers focused on explaining therapeutic models, such as outlining the principles of specific therapies or discussing key learning outcomes, without reporting concrete intervention outcomes or statistical analyses (Bell et al., 2021a; Chadwick, 2003; Cheung and Nguyen, 2012; Engle and Holiman, 2002; Glass, 1995; Greenberg, 2007; Greenberg and Foerster, 1996; Heriot-Maitland et al., 2019; Hill and Norcross, 2023; Holland et al., 2020; Kramer et al., 2020; Lourenço, 2021; Ociskova et al., 2022; Pascual-Leone and Greenberg, 2007; Pos and Greenberg, 2012; Pugh, 2018, 2019; Pugh and Margetts, 2020; Tyson, 1981; Whelton, 2004). We also excluded qualitative studies (Bouffard, 2003; Bulut and Bozo, 2022; Chua et al., 2022; Josek et al., 2023; Pugh et al., 2021; Ramsey, 2012; Steinmann et al., 2017). Excluded studies are characterized as case studies (Argyropoulos and Vlachos, 2024; Cardoso and Eduarda Duarte, 2021; Chastain, 2018; Goldman and Goldstein, 2023; Holmström et al., 2024; Keisari et al., 2023; Lee and Tratner, 2021; Pugh, 2024; Pugh et al., 2023; Sharbanee et al., 2024; Tschan and Goldman, 2024; Tuttle, 1998; Warwar, 2024). We also excluded three studies identified as reviews (Baher, 2023; Greenberg and Pascual-Leone, 2006; Pascual-Leone and Baher, 2023; Pugh, 2017). A further study focused on the development of a questionnaire was also excluded (Singh, 1994). As were studies that did not primarily focus on evaluating the efficacy or effectiveness of the intervention but were primarily focused on therapist factors (Greenberg, 2007; Hall, 2007; Kula et al., 2022; Paivio et al., 2004; Pugh et al., 2021) and client-identified helpful events (Bell et al., 2021b; Holowaty and Paivio, 2012).

Further exclusions were made based on specific circumstances. One study identified as a study protocol was excluded (Kroener et al., 2023), as was a study describing an intervention (Saliani et al., 2024). A further two studies that were categorized as process-outcome and follow-up of other studies were also excluded (Carriere, 2004; Holland et al., 2020). The screening process reduced the total number of eligible articles for the systematic review to 59. However, we decided to include only studies with an RCT design, resulting in a final selection of 22 articles. For a list of non-RCT studies, please refer to Appendix B (see Supplementary material).

**Figure 2:**
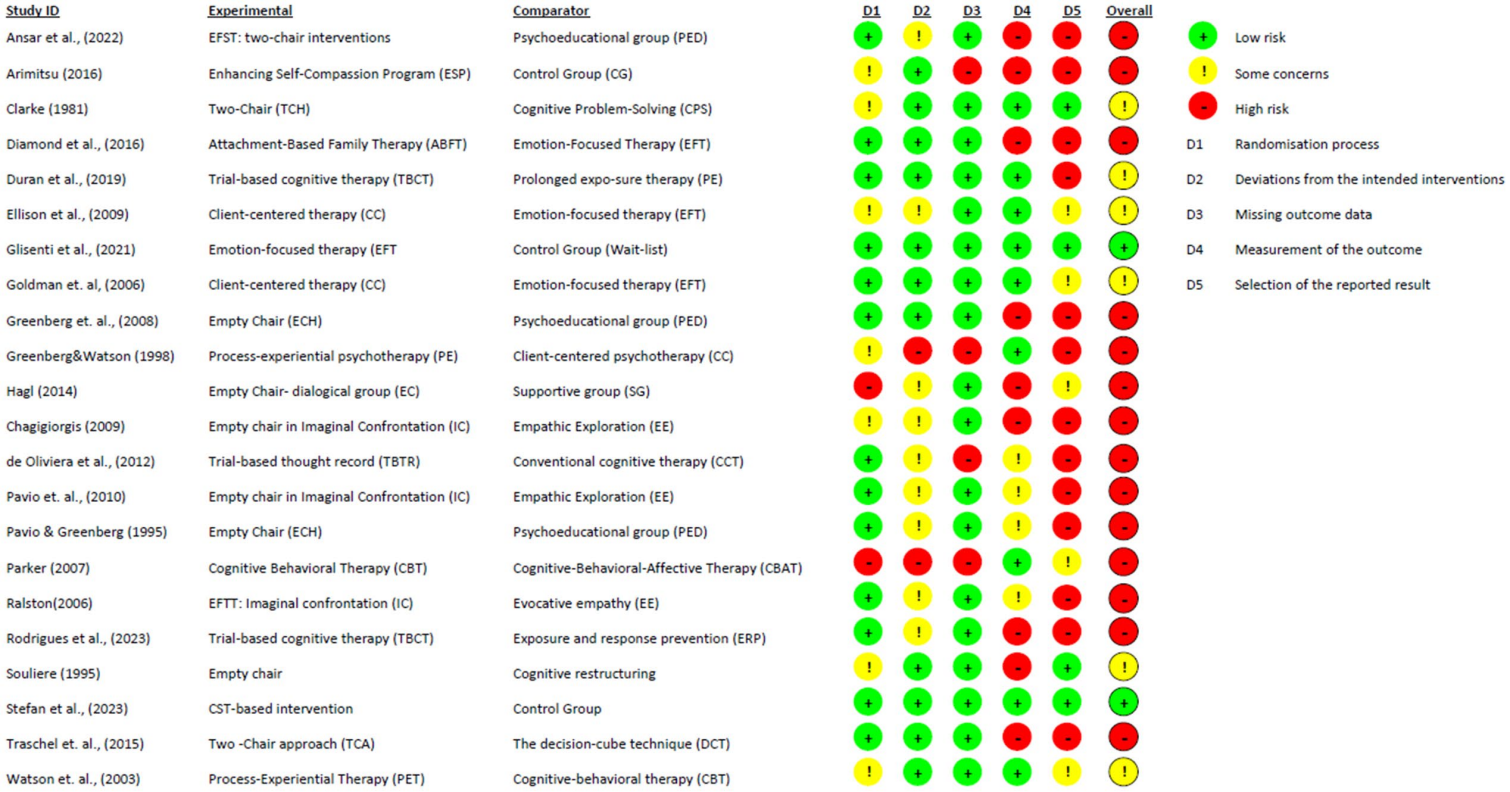
Cochrane risk-of-bias evaluation for randomized controlled trials (ROB-2).

**Figure 3:**
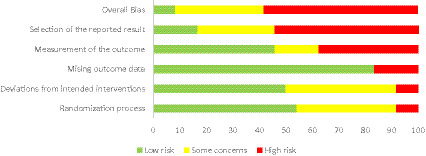
Selection of reported results for RCTs using the ROB-2 tool.

The risk of bias (RoB-2) assessment for the studies is summarized in Figures 2, 3. Only two studies (Glisenti et al., 2021; Stefan et al., 2023) were identified as having a low risk of bias, while 14 were assessed as having a high risk of bias. Additionally, eight studies raised some concerns. The most common sources of bias were incomplete outcome data, inadequate explanations for missing data, and lack of a pre-registered study protocol. Reported biases in the studies included participants’ and therapists’ awareness of the assigned interventions and potential deviations from the intended interventions due to the trial context.

### Characteristics of the studies

In our systematic review, we identified 22 studies targeting various mental health disorders, including depression, eating disorders, obsessive-compulsive disorder (OCD), and social anxiety. Additionally, we included studies that examine career-related psychological processes, such as career decision-making, which focuses on the psychological factors influencing individuals’ career choices and the effectiveness of interventions aimed at facilitating better career decisions. We also identified studies that explore the concept of unfinished business and the impact of childhood trauma. The last category is various family and group therapy interventions.

Across the included RCT studies, Chairwork was implemented in two distinct ways. Most interventions (Ansar et al., 2022; Arimitsu, 2016; Chagigiorgis, 2009; de Oliveira et al., 2012; Diamond et al., 2016; Duran et al., 2020; Ellison et al., 2009; Glisenti et al., 2021; Goldman et al., 2006; Greenberg and Watson, 1998; Hagl et al., 2014; Paivio et al., 2010; Parker, 2007; Ralston, 2006; Rodrigues et al., 2023; Stefan et al., 2023; Watson et al., 2003) incorporated chair-based techniques as one component of a broader psychotherapeutic approach (experiential, cognitive-behavioral, family-based, or compassion-focused frameworks). Chairwork often served as the core therapeutic methodology within these treatments. Although Chairwork was embedded within multimodal treatments, the available data did not allow for isolating the unique contribution of chair-based techniques to overall treatment outcomes. In contrast, a smaller subset of studies implemented Chairwork as a stand-alone method (Clarke, 1981; Greenberg et al., 2008; Paivio and Greenberg, 1995; Souliere, 1995; Trachsel et al., 2012).

In this section, we will report only the results of the identified studies, along with a detailed discussion and interpretation of these findings, which are presented in the following section.

## Mental health issues

### Depression

The first study (Ellison et al., 2009) evaluated the long-term effects of CC (Client-Centered therapy) and EFT (Emotion-focused therapy) on adults with major depression. The researchers followed 43 participants over 18 months, finding that EFT showed better long-term outcomes, with fewer depressive relapses and more weeks without symptoms compared to CC. The study utilized self-report measures and interviews to assess changes in depression, self-esteem, and interpersonal functioning. Emotion-focused therapy demonstrates greater effectiveness in reducing depressive symptoms, improving self-esteem, and reducing general symptom distress and interpersonal problems. Overall, the findings indicate that EFT, with its specific experiential and gestalt-derived interventions, haslong-term effects in maintaining treatment gains and preventing depressive relapse compared to CC, including a higher number of asymptomatic or minimally symptomatic weeks.

The second study (Goldman et al., 2006) examined the effects of adding EFT to the client-centered relationship in the treatment of depression through an RCT with 38 participants who were diagnosed with major depressive disorder. These participants were assigned to one of two groups, one of which used EFT techniques that allow for improved treatment results for major depressive disorders, whereas the second group focused on the traditional relational CC approach. Results showed that both treatments were effective in improving symptoms. The addition of specific Chairwork interventions in the EFT condition resulted in greater overall improvement in mean scores for depressive and general symptoms. Moreover, the study focused on the clinical significance of the treatments, which showed that many participants were assessed as not being depressed following the treatment, with a higher percentage of participants from the EFT group. Although the EFT yielded a greater result overall, the outcome difference between EFT and CC is not that significant, with the former reporting 79% of participants recovering post-test, and the latter 68%. While the EFT group demonstrated a stronger trend toward greater recovery and showed improved outcomes in certain domains, the outcome difference between EFT and CC in reducing depressive symptoms did not reach statistical significance at post-treatment. The use of these empathic, client-centered therapies, Goldman et al. (2006) suggest, has a great impact on symptom reduction and recovery.

Greenberg and Watson (1998) also compared the effectiveness of two psychotherapies, Process-Experiential (PE), later renamed as Emotion-Focused Therapy and Client-Centered therapy, in treating major depression in 34 adults. CC therapy focuses on establishing and maintaining a warm, empathic relationship, while PE therapy combines this with specific techniques such as gestalt interventions to address cognitive-emotional issues. The results showed no significant differences between the two treatments in reducing depressive symptoms at the end of treatment nor at the six-month follow-up. By contrast, PE therapy demonstrated better outcomes at mid-treatment for depression and at the end of treatment by reducing overall symptoms, improving self-esteem, and reducing interpersonal problems. This suggests that adding active interventions to the therapeutic relationship can enhance and accelerate improvement. Both treatments showed significant pre-post effect sizes, indicating that they are effective in treating depression. The study indicates that only the empathic Client-Centered relationship is effective in reducing depressive symptoms and target complaints. At the end of treatment, PE therapy showed greater improvements in self-esteem, interpersonal functioning, and overall distress; though these differences were not evident at the six-month follow-up due to continued improvement in the CC group.

In another study Watson et al. (2003) compared the effectiveness of Process-Experiential Therapy (PET) and Cognitive-Behavioral Therapy (CBT) in treating major depression. Over 16 weeks, 66 clients participated in weekly therapy sessions. Significant improvements were observed in both therapy groups across various measures, including depression levels, self-esteem, general symptom distress, and dysfunctional attitudes. Both therapeutic approaches were found to be highly effective, as indicated by large effect sizes in the reduction of depression symptom severity from baseline to post-treatment (PET: *d* = 1.73; CBT: *d* = 1.69). Additionally, clients in both groups exhibited better coping strategies by the end of treatment. However, PET demonstrated a significantly greater reduction in interpersonal problems compared to CBT. Specifically, PET clients showed significant improvements in being less non-assertive, domineering and controlling, overly accommodating, intrusive, and needy.

The collective evidence consistently suggests that while the establishment of an empathic, client-centered relationship is fundamentally effective, the strategic incorporation of specific experiential techniques, such as Chairwork, contributed to demonstrably greater clinical efficacy (Ellison et al., 2009; Goldman et al., 2006; Greenberg and Watson, 1998). A critical consideration when interpreting these findings is that Chairwork was embedded within a broader, complex treatment framework (EFT/PE). Although this methodological context precludes isolating the specific contribution of Chairwork, the studies consistently showed greater improvements in outcomes such as self-esteem and overall distress when chair-based techniques were included. The active, process-guiding nature of these experiential methods likely functions as a therapeutic accelerant, enhancing the effects of core relational elements.

Interpretation of the reported effect sizes confirms the robust clinical significance of both experiential and cognitive approaches across studies. For instance, the direct comparison between PET and CBT yielded large effect sizes for the reduction of depression severity in both conditions (PET: *d* = 1.73; CBT: *d* = 1.69), indicating that both approaches are highly effective treatments (Watson et al., 2003). However, these studies highlight specific differential advantages for the Chairwork-inclusive, experiential approaches: EFT/PE was found to demonstrate more favorable long-term outcomes in relapse prevention and the maintenance of asymptomatic periods over extended follow-up (Ellison et al., 2009). Furthermore, PET showed a significantly greater reduction in interpersonal problems compared to CBT (Watson et al., 2003). This distinction highlights the unique therapeutic utility of emotionally focused and active processing interventions in resolving complex relational difficulties.

## Other mental health issues

### Eating disorders

Another study (Glisenti et al., 2021) examined the feasibility of using individual EFT to treat Binge-Eating Disorder (BED). The study involved 21 participants aged between 18 and 65 years old, with an average age of 44.52 years, who underwent 12 weekly one-hour EFT sessions over 3 months, with measures including recruitment feasibility, treatment credibility, therapy retention, objective binge episodes and days, and binge-eating psychopathology. The participants were allocated using a block randomization method to either an immediate EFT treatment group or an EFT wait-list control group. The EFT group demonstrated significant improvements in reducing objective binge episodes (eating large amounts of food associated with feelings of loss of control; *d* = 0.99), the number of days with binge episodes (*d* = 1.51), as well as improvements in associated maladaptive behaviors, thinking, and negative emotions (*d* = 1.1), as evidenced by pre-post treatment comparisons. These improvements were maintained at a three-month follow-up, suggesting the potential long-term benefits of EFT. The study also noted a low dropout rate, further indicating the acceptability of EFT among participants.

These findings suggest that EFT, which relies heavily on chair-based emotional processing, is a feasible and effective intervention for Binge-Eating Disorder. The reported Cohen’s *d*-values (0.99, 1.51, and 1.1) represent large effect sizes, indicating a robust clinical impact on both behavioral symptoms and underlying emotional distress. The maintenance of these gains at follow-up supports the utility of experiential methods in securing long-term recovery.

### Obsessive-compulsive disorder (OCD)

A new study by Rodrigues et al. (2023) compared the efficacy of trial-based cognitive therapy (TBCT), a relatively new, empirically validated psychotherapy that utilizes a chair-based “judicial trial” technique to restructure dysfunctional core beliefs, and Exposure and Response Prevention (ERP) for the treatment of OCD. The clinical trial involved 26 randomly chosen individuals who were to receive either TBCT or ERP, with the efficacy being studied over 3 months and consisting of 12 sessions at 3, 6, and 12 months. Initial findings showed that both therapies had significant impacts on the reduction of OCD symptoms and their severity with the gains being observed at the 12-month follow-up. TBCT led to a 47.1% reduction in symptoms from pre-treatment to post-treatment, while ERP resulted in a 43.1% symptom reduction. Additionally, TBCT achieved a 52.6% symptom reduction from pre-treatment to follow-up, and ERP reduced symptoms by 44%. The study suggests that TBCT may be a valid and promising treatment for OCD, as the results persist in the long term, and it offers benefits for patients with anxiety and depression.

The results indicate that Chairwork-based cognitive interventions (TBCT) are comparable in efficacy to the gold-standard treatment (ERP) for OCD. While both treatments were highly effective, the TBCT condition demonstrated a numerically greater percentage reduction in symptoms at both post-treatment and follow-up. This suggests that addressing core beliefs through the experiential “trial” technique may be a valid and promising alternative for OCD, particularly for patients presenting with comorbid anxiety and depression.

### Post-traumatic stress disorder (PTSD)

The study (Duran et al., 2020) compared the effectiveness of two psychotherapies, trial-based cognitive therapy (TBCT) and prolonged exposure (PE), for treating post-traumatic stress disorder (PTSD). It involved 95 patients diagnosed with PTSD, who were randomly assigned to either TBCT or PE. The primary outcome was an improvement in PTSD symptoms, and secondary outcomes included measured change in depression, anxiety, and dysfunctional attitudes. The results showed that both TBCT and PE significantly reduced PTSD symptoms, with no significant difference between the two treatments. However, TBCT was more effective in reducing depressive symptoms (*B* = 3.703, *p* = 0.049). TBCT also had a lower dropout rate compared to PE. The dropout rate was significantly lower in the TBCT group (13.6%) compared to the PE group (33.3%), which was statistically significant (*p* = 0.025). The study suggests that TBCT may be a viable alternative for PTSD treatment and calls for further research to understand its mechanisms of change. It also highlights the high comorbidity of PTSD with other mental health issues and the need for treatments that have lower dropout rates and better patient retention.

These findings highlight the specific utility of Chairwork-integrated cognitive therapy (TBCT) in addressing the complexities of PTSD. While the primary symptom reduction was equivalent to Prolonged Exposure, the differential advantage of TBCT lay in its ability to retain patients in treatment and effectively reduce comorbid depressive symptoms. This suggests that the structured, chair-based restructuring of beliefs may offer a more tolerable and accessible alternative for patients who might otherwise struggle with the demands of pure exposure therapy.

### Social anxiety

de Oliveira et al. (2012) tested the effectiveness of Trial-Based Thought Record (TBTR) and Conventional Cognitive Therapy (CCT) in treating social anxiety disorder (SAD). The TBTR approach focuses on restructuring core beliefs, while CCT uses standard cognitive therapy techniques. The study is a two-arm randomized trial with 36 participants (17 in TBTR, 19 in CCT) diagnosed with generalized SAD according to DSM-IV criteria. The TBTR group engaged in a simulated judicial process to challenge and modify core beliefs, while the CCT group used standard cognitive therapy techniques. The study lasted 12 weeks, with additional follow-up at 12 months. Both TBTR and CCT were found to lead to significant reductions in symptoms of social anxiety and psychiatric distress. However, TBTR was more effective than CCT in reducing fear of negative evaluation (*p* = 0.01 at mid-treatment and *p* = 0.004 at post-treatment), and social avoidance, and distress (*p* = 0.03 at post-treatment).

Contextual Schema Therapy (CST) is a novel psychological treatment that addresses self-criticism and experiential avoidance, two transdiagnostic mechanisms involved in social anxiety. The study (Stefan et al., 2023) aimed to test the efficacy of a brief CST intervention delivered online in group format for individuals with social anxiety symptoms. The intervention included psychoeducation, conceptualization of social anxiety in schema and mode terms, reflection exercises, Chairwork, and imagery techniques. The results showed significant reductions in fear of negative evaluation (*d* = 0.35) and experiential avoidance (*d* = 0.47) in the CST group at the two-week follow-up. Experiential avoidance was found to be a marginally significant mediator of changes in fear of negative evaluation.

The evidence from these studies suggests that Chairwork interventions are effective for Social Anxiety Disorder, whether implemented through a cognitive “trial” framework or a schema mode approach. The de Oliveira et al. (2012) findings are particularly notable, as the active, chair-based component (TBTR) yielded more favorable outcomes in reducing social avoidance compared to standard cognitive therapy. Meanwhile, the effect sizes reported by Stefan et al. (2023), *d* = 0.35 and 0.47 indicate a small-to-medium effect, demonstrating that even brief, online adaptations of Chairwork can successfully target key transdiagnostic mechanisms like experiential avoidance.

### Childhood trauma

Paivio et al. (2010) evaluated the efficacy of two versions of emotion-focused therapy (EFTT) for resolving child abuse trauma: imaginal confrontation (IC) of perpetrators and EFTT with empathic exploration (EE) of trauma material amongst both men and women with a history of childhood maltreatment and various types of abuse. The results showed statistically and clinically significant improvements in symptom distress, self and interpersonal problems, and abuse resolution for both treatment conditions. There were no significant differences between the two versions of EFTT. The study also found that more severe personality pathology negatively influenced some dimensions of the outcome, particularly in EE. Although both treatment options have similar results in improving patient condition, Paivio et al. (2010) observed a significantly higher dropout rate in EFTT-IC at 20% compared to EFTT-EE at 7%. Nonetheless, despite the different attrition rates, the study observed that patients treated with both conditions maintained their treatment gains at follow-up, ranging from 6 to 18 months. The study concludes that both versions of EFTT are effective in treating complex PTSD resulting from childhood trauma. While the IC procedure may offer some benefits over EE, including a higher rate of clinically significant change, the EE procedure has a lower dropout rate and could be a less stressful alternative for certain clients.

Chagigiorgis (2009) replicated and extended the research findings reported by Paivio et al. (2010) by studying the effects and treatments of childhood trauma using Emotion Focused Trauma Therapy (EFTT): one with Imaginal Confrontation (IC) using an empty chair, and another with Empathic Exploration (EE) where clients focus on traumatic experiences and imagine the other person in their “mind’s eye.” The study found that both IC and EE promoted therapeutic processes consistent with EFTT principles and led to improvements in various dimensions, including reductions in symptom distress, improvements in affect regulation and interpersonal skills, and resolution of issues with perpetrators of abuse. It also highlights the importance of emotional engagement and psychological contact with trauma material for achieving positive outcomes. Client-reported emotional engagement during IC sessions was found to significantly predict the resolution of abuse issues (*R*^2^ = 0.50, *p* < 0.05). For the EE condition, higher observed emotional engagement significantly predicted a reduction in subsequent interpersonal problems (*R*^2^ = 0.35, *p* < 0.01) and a reduction in anxiety (*R*^2^ = 0.41, *p* < 0.05). The study presents a detailed analysis of client engagement, including the use of ‘I’ language, spontaneous elaboration of feelings, and dialogue related to abuse work. Engagement is categorized into levels, with “full engagement” indicating vivid descriptions of trauma, emotional arousal, and a focus on abuse work.

Lastly, Ralston’s (2006) study is part of a larger comparison of two versions of EFTT for adult childhood abuse survivors. The study aimed to explore the similarities and differences between IC and EE by examining key client processes, including experiencing, emotional arousal, and therapeutic alliance. Both IC and EE interventions in EFTT were generally equivalent in terms of effectiveness for adult survivors of childhood abuse. Both treatments produced a significant level of positive change in outcome measures across multiple domains. The study also revealed meaningful patterns of similarities and differences in client processes between IC and EE, with some findings supporting the hypotheses and expectations of the researchers. For instance, the therapeutic alliance was found to be equally strong in both groups, and the quality of the alliance predicted improved outcomes in both IC and EE, with a broader impact in the EE group. Additionally, the study found that engagement with trauma material increased over time in the IC group, suggesting that clients in this condition overcame initial difficulties with avoidance, emotion regulation, or social anxiety.

While Chairwork (Imaginal Confrontation) is as effective as imaginal exposure (Empathic Exploration) in reducing trauma symptoms, it presents a higher demand on the client. The evidence indicates equivalent efficacy between the two modalities regarding symptom distress and abuse resolution (Paivio et al., 2010; Ralston, 2006). However, the substantially higher dropout rate in the Chairwork condition (20% vs. 7%) suggests that the intense emotional arousal and confrontation inherent to the empty-chair technique may be more difficult for some clients to tolerate. This implies that while Chairwork is a potent intervention, clinicians must carefully assess client readiness and potentially utilize Empathic Exploration as a gentler alternative for improved retention.

Chairwork appears to facilitate a distinct trajectory of engagement with traumatic material. Although the outcomes were similar, Ralston (2006) noted that engagement increased over time in the Chairwork condition, indicating that the technique effectively helps clients overcome avoidance and social anxiety related to the trauma. This aligns with Chagigiorgis’s (2009) finding that full engagement, characterized by vividness and arousal, is crucial for change. Therefore, the primary utility of Chairwork in trauma may not be in superior statistical outcomes compared to other exposure methods, but in its specific ability to deepen emotional processing and overcome avoidance in clients who are able to remain in treatment.

### Unfinished business

Greenberg et al. (2008) compared the effectiveness of EFT using the empty-chair dialogue versus psychoeducation groups (PG) in facilitating forgiveness and the resolution of emotional injuries. The study found that EFT was more effective than PG treatment in promoting forgiveness, resolving emotional injuries, and reducing general symptoms. Specifically, the EFT group exhibited higher levels of forgiveness-related gains and greater symptom reduction compared to the PG group. The study also found that clients who reported forgiving their injurer additionally reported that they were able to let go of distressing feelings and unmet needs associated with the injury. However, some individuals let go of their injuries, feelings, and needs without forgiving the injurer, suggesting that letting go of negative emotions may be a necessary but not sufficient step for forgiveness. Based on the findings and observations, the study shows that EFT is an effective approach for promoting forgiveness and the resolution of emotional injuries and emphasizes the need for tailored therapeutic interventions, which look at and address the underlying emotional processes that lead to forgiveness.

Similarly, Paivio and Greenberg (1995) investigate the effectiveness of experiential therapy using Empty-Chair Dialogue Intervention (ECH) compared to psychoeducational group (PED) in resolving unresolved feelings toward a significant other, termed “unfinished business.” Experiential therapy using ECH was found to be significantly more effective than PED in resolving unfinished business related to significant others. The ECH therapy led to clinically meaningful improvements for most clients, including significant reductions in symptoms and interpersonal distress, increased self-acceptance, and decreased perceptions of hostility in relationships. These gains were largely maintained at 4-month and 1-year follow-ups.

The evidence indicates that active, experiential processing via Chairwork is significantly more effective than cognitive psychoeducation for resolving complex relational injuries. Both Greenberg et al. (2008) and Paivio and Greenberg (1995) demonstrate that while understanding the trauma (psychoeducation) may offer some benefit, the enactment and expression of emotion provided by the empty-chair dialogue are necessary to achieve substantial symptom reduction and resolution of “unfinished business.”

The Empty Chair technique facilitates deep emotional shifts, specifically “letting go” and forgiveness, that are robust and durable over time. The maintenance of gains at the 1-year follow-up in the Paivio and Greenberg (1995) study supports the validity of Chairwork as a mechanism for structural emotional change rather than just temporary symptom relief. Furthermore, the nuance identified by Greenberg et al. (2008), that one can “let go” without necessarily forgiving, highlights the precision of the empty-chair technique in allowing clients to process negative affect (anger, hurt) independently of their decision to reconcile with or forgive the perpetrator.

### Unfinished business (maladaptive anger)

Souliere (1995) addresses methodological issues in previous studies on the topic of anger that employ longer treatment sessions, and a comprehensive battery of outcome measures, and hypothesizes that the Gestalt empty chair dialogue would be more effective in achieving anger resolution regarding lingering feelings toward a significant other compared to cognitive restructuring using RET. The former focuses on the importance of experiencing and expressing primary emotions to resolve unfinished business, whilst the latter considers emotions as post-cognitive and irrational and looks at modifying the person’s belief system through cognitive restructuring. In conclusion, the study did not find evidence to support the hypothesis that Gestalt empty chair dialogue is more effective than cognitive restructuring in resolving lingering angry feelings. The research highlights the importance of addressing and expressing anger in a healthy, constructive manner for personal growth and improving relationships.

The study (Parker, 2007) took the form of a randomized controlled trial with a 1-month follow-up, involving 20 participants, 10 of whom were assigned to the Cognitive Behavioral Therapy (CBT) group and 10 to the Cognitive Behavioral Affective Therapy (CBAT) group. The results show that both treatments were effective in reducing anger, and the reduction was still in evidence at the one-month follow-up. Between-group comparisons showed no significant differences, but the observed trend suggested that the CBAT group experienced a slightly greater reduction in anger intensity and other anger variables at post-treatment and follow-up. The findings suggest that CBAT, an integrative approach, may be more effective than traditional CBT at addressing problematic anger. However, further research with larger sample sizes and additional analyses is needed to support these findings and explore the mechanisms of change.

Unlike comparisons with psychoeducation, Chairwork-based interventions appear to be statistically equivalent to active cognitive treatments (CBT/RET) for the reduction of maladaptive anger. Both Souliere (1995) and Parker (2007) found that both expressing emotion (Chairwork) and restructuring thoughts (CBT) lead to significant symptom reduction. This suggests that “unfinished business” can be effectively resolved through different therapeutic pathways, either by processing the affective experience directly or by modifying the cognitive appraisals fueling the anger.

While statistical significance was not achieved, there is a tentative indication that integrating experiential components into cognitive frameworks (CBAT) may offer incremental benefits. Parker (2007) noted a trend favoring the integrative approach, hinting that the addition of affective processing might enhance standard CBT outcomes. However, this interpretation must be viewed with caution due to the low statistical power resulting from small sample sizes (*n* = 20), which likely precluded the detection of moderate between-group differences.

### Career decision-making

The researcher Clarke (1981) conducted a study using these two approaches with participants who were experiencing career-related conflict. The 48 participants were randomly assigned to either the Gestalt Two-Chair experiment (TC) group, the Cognitive Problem-Solving (PS) group, or a control group, where each group contained 16 participants. The aim was to evaluate the impact of each counseling method against depth of experience, awareness, conflict resolution, and behavior change. The Two-Chair Experiment, an affective counseling approach, was found to be significantly more effective at reducing indecision than Problem-Solving, a cognitive counseling approach. Moreover, the results indicate that the affective approach led to a greater reduction in indecision than the cognitive approach.

This study provides a critical counter-narrative to the assumption that decision-making is a purely rational or cognitive task. The finding that Chairwork (an affective intervention) outperformed Problem-Solving (a cognitive intervention) suggests that career indecision is often fueled by underlying emotional conflicts or “splits” rather than a simple lack of analytical skills or information. Consequently, addressing the felt sense of the conflict through the Two-Chair enactment allows for a deeper resolution of ambivalence than cognitive weighing of pros and cons alone. This highlights the specific utility of Chairwork in non-clinical, counseling settings where ambivalence is the primary barrier to action.

### Partnership ambivalence

A study by Trachsel et al. (2012) compared the effectiveness of two very brief interventions, the two-chair approach (TCA) and the decision-cube technique (DCT), in reducing partnership ambivalence. Results indicated that both interventions led to a significant reduction (*d* = 1.54) in partnership ambivalence, with no significant difference between the TCA and DCT conditions at post-intervention or 4-month follow-up assessment. However, participants who had been ambivalent for more than a year showed a greater reduction in ambivalence with the DCT (*d* = 0.61) compared to the TCA at post-assessment, suggesting that the DCT might be more effective for individuals with more persistent ambivalence. The study also examined the relationship between partnership ambivalence and psychological wellbeing, finding that ambivalence was positively associated with depressive symptoms and distress, and negatively associated with life satisfaction. Interestingly, depressive symptoms decreased significantly over time in the DCT condition (*d* = 0.68), but not in the TCA condition. Mechanisms of change, such as processual activation and clarification, were also investigated. The TCA was found to be associated with higher levels of processual activation (*d* = 0.72), but no significant difference in clarification was observed between the two interventions.

Both experiential and cognitive brief interventions are highly effective for resolving relationship ambivalence. The large effect size (*d* = 1.54) shared by both groups indicates that the active ingredient in resolving ambivalence may be the focused attention and processing of the conflict, regardless of whether the modality is emotional (Chairwork) or rational (Decision Cube).

While Chairwork successfully generates higher emotional activation (*d* = 0.72), this heightened arousal may not always be the optimal pathway for chronic decision-making conflicts. The finding that the cognitive DCT was more effective for participants with long-term ambivalence and for reducing associated depressive symptoms suggests that when a client remains “stuck” for extended periods, a structured cognitive framework may provide better containment and relief than further emotional intensification. This implies a differential indication: Chairwork may be best for accessing suppressed emotion, while cognitive tools may be superior for organizing a chaotic or chronic decision process.

### Family-based interventions

The study by Ansar et al. (2022) highlights the efficacy of Emotion-Focused Skills Training (EFST) in reducing internalizing and externalizing symptoms in children through parental training. The study included parents of 236 children with clinical range symptoms and randomly assigned them to two groups: one received experiential EFST and the other psychoeducational EFST. Derived from Emotion-Focused Therapy, EFST led to significant reductions in parent-reported externalizing (*d* = 1.0) and internalizing (*d* = 0.9) symptoms, and teacher-reported externalizing symptoms (*d* = 0.4). However, it did not significantly impact teacher-reported internalizing symptoms (*d* = 0.2). There were no significant differences between the two conditions, although the experiential condition seemed to have a better outcome.

A study by Diamond et al. (2016) compared the effectiveness of Attachment-Based Family Therapy (ABFT) and EFT in treating unresolved anger in young adults toward their parents. It found that both therapies led to significant decreases in unresolved anger, state anger, attachment anxiety, and psychological symptoms. However, only ABFT was associated with decreases in attachment avoidance. The study also found that greater amounts of productive emotional processing predicted greater reductions in psychological symptoms across both treatments.

Interventions that engage parents in emotional processing yield large effects on child symptom reduction. The large effect sizes reported by Ansar et al. (2022) for parent-reported symptoms suggest that equipping parents with emotional skills (often practiced via chair enactments in EFST) is a highly potent intervention. Although the difference between experiential and psychoeducational modes was not statistically significant, the trend favoring the experiential group aligns with previous findings suggesting that active enactment may deepen the acquisition of caregiving skills.

While Chairwork (EFT) and direct family therapy (ABFT) are equally effective for resolving anger, direct interaction may be superior for reducing avoidance. Diamond et al. (2016) provide a crucial distinction: the empty chair (EFT) is sufficient for processing the emotion of anger and anxiety, but the “live” presence of the parent (ABFT) appears necessary to overcome attachment avoidance. This suggests a specific clinical indication: when the primary therapeutic goal is to bridge an emotional distance or cut-off (avoidance), in-vivo family work may be preferable to individual Chairwork. However, for symptom reduction and anger processing, the individual Chairwork format remains a robustly effective alternative.

### Group-based interventions

Arimitsu (2016) investigated the effectiveness of the Enhancing Self-Compassion Program (ESP) among Japanese individuals with low self-compassion. The aim was to establish whether the ESP could improve self-compassion and related psychological outcomes in a culture that is typically more self-critical and less self-compassionate. Forty participants were divided into an ESP group and a wait-list control group. The study demonstrated that the ESP program significantly improved self-compassion, and self-esteem and reduced negative automatic thoughts, anxiety, depression, and shame among participants with substantial effect sizes ranging from 0.91 to 1.51. Qualitative feedback further supported the program’s positive impact, highlighting its benefits in fostering compassion, acceptance, and motivation to change. However, some participants experienced difficulties with certain aspects of the program, particularly the meditation exercises. Overall, the ESP program appears to be a promising intervention for enhancing self-compassion and related psychological outcomes in a highly self-critical culture.

Hagl et al. (2014) discuss the effectiveness of Chairwork, specifically the empty-chair method, in the treatment of symptoms related to traumatic loss in Bosnian women. The study involved 119 women who had lost their husbands during the Bosnian war. The study compared a dialogical group approach using empty-chair exposure to a supportive group approach where participants talked about their husbands. Both interventions resulted in significant improvement in post-traumatic symptoms, general mental health, and grief reactions. The dialogical exposure group showed moderate improvements (*d* = 0.56), while the supportive group showed small improvements (*d* = 0.34). However, the dialogical exposure group was found to be superior in reducing traumatic grief and post-traumatic avoidance symptoms. Dialogical exposure was superior as far as traumatic grief (*d* = 0.37) and post-traumatic avoidance (*d* = 0.73) post-treatment are concerned. Overall, the short-term dialogical exposure group treatment was moderately effective in treating traumatically bereaved women.

Group-based Chairwork appears to offer a distinct advantage over standard support groups in reducing pathological avoidance. The findings by Hagl et al. (2014) provide a compelling differentiation: while supportive conversation helps with general distress (*d* = 0.34), the active enactment of the empty-chair dialogue (*d* = 0.56) is necessary to break through the avoidance mechanisms associated with traumatic grief. The notably higher effect size for avoidance reduction (*d* = 0.73) suggests that the experiential nature of Chairwork forces a confrontation with the trauma that narrative support alone may not achieve.

Chairwork-based interventions demonstrate robust efficacy across diverse cultural contexts, with effect sizes appearing particularly large in interventions targeting self-criticism. Arimitsu’s (2016) study in Japan reported large effect sizes (*d* > 0.90) for self-compassion and shame reduction. This suggests that externalizing the “internal critic” via Chairwork may be an especially potent mechanism in cultures where shame and self-criticism are deeply internalized. Collectively, these studies confirm that the efficacy of Chairwork is not limited to individual Western therapy but is transferable to group formats and non-Western populations.

## Discussion

The primary objective of this systematic review was to evaluate the therapeutic efficacy of treatments that incorporate Chairwork, as demonstrated in randomized controlled trials (RCTs). The analysis of the 22 included studies provides robust empirical support for the utility of Chairwork across a diverse spectrum of clinical presentations, including depression, childhood trauma, unfinished business, and a heterogenous category of disorders comprising OCD, PTSD, social anxiety, and eating disorders. A distinct finding of this review is the versatility of Chairwork’s implementation: while predominantly utilized as a core experiential component within broader psychotherapeutic frameworks (e.g., Emotion-Focused Therapy, Trial-Based Cognitive Therapy), it also demonstrated efficacy as a stand-alone intervention in a smaller subset of studies. Across these distinct applications, reported effect sizes (Cohen’s *d*) varied, ranging from small (*d* = 0.20) to large (*d* = 1.73), generally indicating a high level of therapeutic potency for active emotional enactment. The following sections provide a detailed synthesis and interpretation of these findings.

### Depression

All three studies (Ellison et al., 2009; Goldman et al., 2006; Greenberg and Watson, 1998) investigated and compared the outcomes achieved by Client-Centered therapy (CC) and Emotion-Focused Therapy (EFT). Clients met the diagnostic criteria for depressive disorder. The combined results indicate that EFT produced significantly better outcomes regarding depressive and general symptom distress, self-esteem, and interpersonal functioning at the end of treatment (Goldman et al., 2006; Greenberg and Watson, 1998). Adding emotion-focused interventions such as Chairwork to the Client-centered approach enhanced the treatment’s efficacy. Clients who received EFT through Chairwork experienced a significant reduction in depressive symptoms.

Moreover, two of the identified studies (Greenberg and Watson, 1998; Ellison et al., 2009) also investigated whether reductions in depressive symptomatology were maintained after treatment. Greenberg and Watson (1998) measured clients’ levels of depressive symptoms 6 months after the treatment ended. They found no statistically significant change in depressive symptom level (BDI; Beck et al., 1961) compared to the end-of-treatment measurements. The study by Ellison et al. (2009) measured depressive symptom levels at 6 and 18 months after treatment ended. The authors of the study showed that depressive symptom rates (BDI; Beck, 1961) were statistically significantly lower at both 6 and 18 months after treatment compared to their baseline rates. Thus, the results of the studies suggest that therapeutic change is stable over time when EFT is used to treat depression (Greenberg and Watson, 1998) and has long-term efficacy (Ellison et al., 2009). Additionally, Watson et al. (2003) compared the effectiveness of Emotion-Focused Therapy (EFT) and Cognitive Behavioral Therapy (CBT) in the treatment of depression. Both therapeutic approaches were found to be highly effective, as indicated by the large effect sizes in the reduction of depression symptom severity from baseline to post-treatment. There were no significant differences in efficacy between the two therapies. Chair-based interventions can effectively improve clients’ overall interpersonal functioning.

The findings from all four studies (Ellison et al., 2009; Goldman et al., 2006; Greenberg and Watson, 1998; Watson et al., 2003) provide evidence supporting the efficacy of Emotion-Focused Therapy in treating Major Depressive Disorder, outperforming Client-Centered Therapy and Cognitive Behavioral Therapy. This consistency suggests that EFT, particularly with Chairwork, has the potential to become the preferred therapeutic approach for individuals with depression. While the results are promising, the small sample size limits the generalizability of the findings. Additionally, relying on self-reported depression measures may introduce bias. Future research is needed with larger, more diverse participant groups and objective depression assessments.

### Eating disorders

A pilot study (Glisenti et al., 2021) tested the efficacy of individual EFTs in the treatment of eating disorders, specifically binge eating disorder (BED). The findings suggest that existing CBTs may have limited effectiveness when used to treat binge eating disorder, as they often fail to adequately address the role of negative emotions. The studies reviewed indicate that a focus on emotional processing and resolution through interventions like Chairwork may be more effective in reducing binge eating episodes and associated symptoms. This points to the importance of targeting the underlying emotional factors that contribute to binge eating, rather than solely relying on behavioral and cognitive strategies. By addressing the emotional experiences and interpersonal difficulties that drive binge eating, treatment approaches may be better equipped to produce more meaningful and lasting outcomes for individuals struggling with this disorder. A limitation of this pilot RCT is the relatively small sample size, the high percentage of women (80%), and the fact that all the participants received EFT from a single therapist. This may have led to therapist effects and reduced the generalizability of the findings.

### Obsessive-compulsive disorder (OCD)

A new study (Rodrigues et al., 2023) compared the efficacy of Trial-based Cognitive therapy (TBCT) and Exposure and Response Prevention (ERP) for the treatment of OCD. The results showed that both TBCT and ERP significantly reduced the severity of OCD symptoms with large effect sizes. These improvements were maintained at the 12-month follow-up assessment. Empty Chair is used during trial-based thought record (TBTR) and aims to reduce attachment to dysfunctional negative core beliefs (CBs) and the intensity of accompanying emotions. It allows for experiential exploration and the restructuring of cognitive beliefs and emotions. The study suggests that the inclusion of the Empty Chair technique during TBTR might boost efficacy for reducing attachment to CBs and the intensity of the accompanying emotions.

### Post-traumatic stress disorder (PTSD)

The study by Duran et al. (2020) investigated the use of prolonged exposure (PE) and trial-based Chairwork interventions. Prolonged exposure involved clients vividly imagining and narrating aloud the details of their traumatic experiences while performing breathing exercises to modulate the resulting emotional arousal. Trial-based Chairwork was employed to restructure the distorted cognitions thought to underlie the shame and sadness experienced by clients in response to their traumatic events. The findings revealed no significant differences in the improvement in dysfunctional attitude between the two treatment conditions.

Support for these experiential approaches is further elucidated by Kellogg (2023), who formalized the “Four Dialogues” method in Chairwork psychotherapy. Kellogg posits that the technique of “Telling the Story” functions as a form of controlled emotional exposure, while “Imaginal Confrontation” facilitates the external processing of intense emotions, such as anger and grief. This dialogue process ultimately aims to help clients reach a “redecision” regarding self-limiting or dysfunctional life narratives (Kellogg, 2023). Complementing this theoretical framework, Prasko et al. (2025) have demonstrated the clinical utility of these methods in populations with complex trauma. Their case studies indicate that the integration of Imagery Rescripting with Chairwork can effectively mitigate symptoms associated with transgenerational trauma (Prasko et al., 2025). While Duran et al. (2020) found no significant differences between prolonged exposure and trial-based Chairwork in reducing dysfunctional attitudes, the combined use of Imagery Rescripting and Chairwork may provide clients with a mechanism to actively transform maladaptive family patterns into more adaptive coping strategies, supporting the utility of focused experiential processing for complex trauma histories.

### Social anxiety disorder (SAD)

Social anxiety disorder (SAD) is one of the most common mental disorders and can be chronic and debilitating. While CBT is effective, a significant number of patients do not improve (Kessler et al., 1999). TBTR is a new approach that targets and restructures patients’ core beliefs, which are thought to underlie and maintain social anxiety symptoms (de Oliveira, 2008). Preliminary findings from the study (de Oliveira et al., 2012) suggest that TBTR is at least as effective as Conventional Cognitive Therapy (CCT) in reducing symptoms of SAD and may be particularly effective in reducing fear of negative evaluation, social avoidance, and distress. These results align with those of a pilot study by Shahar et al. (2017), which supported the efficacy of an individualized form of EFT in treating social anxiety. Following EFT treatment, significant improvement in symptoms was observed, with positive changes remaining stable at 6- and 12-months post-treatment.

Furthermore, a study by Stefan et al. (2023) examined the efficacy of Contextual Schema Therapy (CST) as a psychological treatment for social anxiety. CST addresses self-criticism and experiential avoidance, two transdiagnostic mechanisms involved in social anxiety. The study tested a brief CST intervention delivered online in group format and found it effective in reducing social anxiety symptoms. Notably, the reduction in experiential avoidance was identified as a potential mechanism of change. In a comparative study Thew et al. (2022) emphasized traditional cognitive therapy techniques and explored the effectiveness of internet-delivered cognitive therapy (iCT) for SAD. This study found that iCT significantly reduced symptoms of social anxiety, with participants showing improvement in social interaction and a reduced fear of negative evaluation. The use of an online format for therapy in both studies highlights the growing feasibility and acceptability of digital interventions for SAD.

Together, the studies (de Oliveira et al., 2012; Stefan et al., 2023) highlight the potential of both TBTR and CST as viable treatments for SAD. TBTR’s effectiveness in addressing specific symptoms such as fear of negative evaluation and social avoidance sits alongside the long-term stability of symptom reduction. Meanwhile, CST offers a promising approach by targeting self-criticism and experiential avoidance, with the added benefit of online group delivery. The convergence of these findings suggests that a variety of therapeutic approaches can be successful in managing social anxiety, offering flexibility in treatment options based on individual patient needs and preferences.

### Childhood trauma

We identified three studies (Paivio et al., 2010; Chagigiorgis, 2009; Ralston, 2006) that examined the efficacy of treating childhood trauma using Emotion Focused Trauma Therapy (EFTT), a short-term treatment approach grounded in experiential therapy theory and research on emotion-focused therapy. This type of therapy consists of 16–20 hourly sessions per week involving four phases and tasks that create a therapeutic alliance, overcome self-related difficulties, resolve issues related to abuse and neglect, and restructure relationships. The clients were women and men with different types of childhood abuse (abuse, neglect, etc.) The studies compared and assessed the effectiveness of two versions of EFT: one with Imaginal Confrontation (IC), and one with Empathic Exploration (EE). IC involves clients directly confronting their trauma memories, while EE focuses on emotional engagement with trauma material.

The study (Paivio et al., 2010) concluded that both versions of EFTT are effective in treating complex PTSD resulting from childhood trauma. Paivio et al. (2010) study was followed up by Ralston (2006), and both emphasized the role of emotional processing and resolution facilitated by Chairwork, which led to improved psychological wellbeing. Clients were randomly divided into two groups: 20 clients received EFT including IC with the imagined abuser, while the other 20 clients used empathic exploration (EE) to process traumatic content. Chagigiorgis (2009) further highlighted the sense of empowerment and emotional relief experienced by participants, suggesting that Chairwork not only reduces symptoms but also promotes a sense of control and mastery over traumatic experiences.

The studies reviewed show that both treatments led to significant improvements in global distress, trauma symptoms, and interpersonal problems with positive changes sustained months after treatment. The follow-up period ranged from 6 to 18 months, with an average length of approximately 1 year. The relationship between emotional arousal and symptom reduction was statistically significant, indicating that higher emotional engagement during therapy sessions correlates with better outcomes (Chagigiorgis, 2009). These findings align with previous research on experiential therapies (e.g., Paivio and Nieuwenhuis, 2001), which emphasizes the importance of engaging clients in active, expressive processes to resolve emotional conflicts and integrate traumatic memories. The results support the theoretical foundations of Chairwork, which posit that giving voice to different parts of the self and directly confronting traumatic memories can lead to significant therapeutic gains (Kellogg and Garcia Torres, 2021).

A significant limitation of these studies is that participants in both groups, clients received treatment through EFT, but with different therapeutic techniques. There was no control group of clients receiving treatment through a different approach or with no intervention. Nonetheless, EFT for trauma treatment is likely to be effective. Further studies examining EFT for trauma treatment and comparing it with other psychotherapeutic approaches would be useful. If support is found for the efficacy of EFT for trauma treatment, that would improve the range of interventions in this treatment area.

### Unfinished business

In this review, we identified four studies examining the use of chair-based experiential interventions for addressing unresolved emotional experiences commonly described as unfinished business. Two of the included studies focused on emotional injuries involving significant others, and two examined maladaptive anger rooted in unresolved relational conflict.

The studies (Greenberg et al., 2008; Paivio and Greenberg, 1995) examined the comparative effectiveness of empty-chair work and psychoeducation in addressing unresolved emotional injuries involving significant others. These emotional injuries were characterized by the researchers (Paivio and Greenberg, 1995) as significant interpersonal harms resulting in persistent feelings of hurt, anger, and betrayal, often stemming from experiences of abandonment, betrayal, or violation by important figures such as parents, partners, friends, or authority figures. The studies emphasized that these injuries encompass both emotional and interpersonal dimensions and focused on interventions to facilitate the resolution of injury by alleviating distress and promoting forgiveness. Additionally, a study by Greenberg et al. (2008) found that the empty-chair work intervention was significantly more effective than other conditions in reducing participants’ overall symptoms by the end of treatment and the 3-month follow-up assessment. Similarly, the study by Paivio and Greenberg (1995) demonstrated that the empty-chair work intervention was significantly more effective in reducing general symptoms and interpersonal distress compared to other conditions, and the improvements were still evident up to 1 year after treatment. These studies highlighted the importance of addressing unresolved emotional experiences with significant others in therapy. The researchers emphasized the role of emotional arousal and the expression of previously suppressed primary emotions in the process of resolving unfinished business. Building upon insights, Greenberg and Malcolm (2002) presented a model specifying the essential components of the resolution, including the expression of previously unmet interpersonal needs, a shift in the view of the other, and resolution. The findings of this research indicated that clients who were able to express intense primary emotions, such as anger and sadness, assert previously unmet needs, and shift their view of the other, and achieved resolution, demonstrated significantly better treatment outcomes.

### Unfinished business (maladaptive anger)

Lingering unresolved anger toward a significant other is considered particularly problematic, as unexpressed angry feelings can impact daily functioning and behavior. These feelings may resurface when the individual thinks about, remembers, or encounters the person in question. Despite attempts to resolve these emotions, they persist and may lead to discomfort and tension (Pascual-Leone et al., 2013). The study by Souliere (1995) aims to address these unresolved anger problems through therapeutic interventions, such as Gestalt empty chair dialogue and cognitive restructuring, to help individuals process and resolve these lingering emotions. Similarly, Parker (2007) presents an analysis of the comparative efficacy of Cognitive Behavioral Therapy (CBT) and Cognitive-Behavioral-Affective Therapy (CBAT) in treating problematic anger, finding both treatments to be effective with results being sustained over time, but with no significant differences between them.

These studies discuss the assessment of these anger problems, the therapeutic techniques used to address them, and the outcomes of the interventions. They also highlight the importance of fully understanding and experiencing these emotions to develop a comprehensive understanding of events and situations, and to choose appropriate responses. Findings suggest that both affective and cognitive techniques may be equally effective in dealing with unresolved anger toward a significant other and that changes in one domain may produce changes in the other.

### Career decision-making

Only one eligible randomized controlled study examined the use of Chairwork techniques in the context of career decision-making (Clarke, 1981). Clarke’s (1981) research is grounded in the understanding that intrapersonal conflicts can significantly inhibit decision-making processes. The Gestalt Two-Chair (TC) technique focuses on resolving conflicts by heightening awareness through a dialogical process between the conflicting parts. At the same time, Cognitive Problem-Solving (PS) emphasizes a structured, rational approach to problem resolution involving steps such as general orientation, problem definition, generation of alternatives, decision-making, and verification. The results showed that individuals with high theoretical knowledge benefitted more from career exploration programs than those with low theoretical knowledge. Additionally, the study provides important insights into the approaches counselors can implement. One observation was that counselors focusing on behavioral interventions would strongly benefit from adding an affective approach to their repertoire. The theoretical relevance of Clarke’s findings is supported by research Greenberg and Webster (1982), who examined the mechanisms underlying effective two-chair work in decision-making contexts. Their study provides insight into the in-session processes that may explain why experiential approaches can be beneficial. They identified three critical markers of productive conflict resolution: the expression of internal criticism, the articulation of feelings and wants, and a softening of the self-critical stance. Clients classified as “resolvers” based on these process indicators demonstrated substantial reductions in decisional conflict and anxiety, along with greater improvements in goal attainment and behavior change, compared to “non-resolvers.” These findings suggest that emotional differentiation and the transformation of internal criticism may be key therapeutic mechanisms activated during experiential Chairwork.

Both studies (Clarke, 1981; Greenberg and Webster, 1982) underline the significance of the therapeutic process in achieving positive outcomes. Clarke (1981) indicated that the Gestalt approach’s emphasis on emotional and experiential engagement leads to deeper awareness and conflict resolution. However, the study is the only randomized controlled trial examining the use of two-chair dialogue in the context of career decision-making. The current evidence base remains limited. Although the findings provide valuable initial insight into how experiential dialogue may support the resolution of decisional conflict, further research is needed to strengthen and extend these conclusions. Future studies employing larger and more diverse samples, as well as additional randomized designs, would help clarify the robustness and generalizability of these effects.

### Partnership ambivalence

Partnership ambivalence refers to a state of uncertainty or indecision about whether to continue or end a romantic partnership. It involves a conflict within the individual who is unable to make a clear decision about the future of the relationship. This ambivalence can be a significant source of psychological distress and can impact the person’s overall wellbeing and life satisfaction (Zoppolat et al., 2024). A study by Trachsel et al. (2012) found that both the Two-Chair Approach (TCA) and the Decision-Cube Technique (DCT) were effective in reducing partnership ambivalence, with the DCT showing particular promise for individuals with more persistent ambivalence and for reducing depressive symptoms, while the TCA was more effective at activating emotional processes during the intervention.

Several limitations of the study should be considered when interpreting these findings. The study’s exploration of the mechanisms of change raises important questions. The TCA was associated with higher levels of processual activation, indicating that it may engage clients more deeply in the emotional and cognitive processes related to their ambivalence. Despite this, the lack of a significant difference in clarification between the two interventions suggesting that both methods are equally effective in helping clients understand their ambivalence. Also, the brief nature of the interventions and relatively short follow-up period (3 months) may not have captured the full extent of their long-term efficacy. Additionally, the reliance on self-reported measures may have introduced bias, as participants’ perceptions of their ambivalence and wellbeing may be influenced by factors not controlled for in the study.

### Family-based interventions

This review identified three distinct RCTs examining the efficacy of group and family interventions utilizing Chairwork. Each study examines a different therapeutic approach: Emotion-Focused Skills Training (EFST) for parents, Emotion-Focused Therapy (EFT) vs. Attachment-Based Family Therapy (ABFT) for unresolved anger, and the Enhancing Self-Compassion Program (ESP) for individuals with low self-compassion.

Ansar et al. (2022) examined the efficacy of Emotion-Focused Skills Training (EFST), which is a 12-week parental program aimed at improving mental health problems in children and adolescents. EFST is designed to enhance parents’ capacity to respond adaptively to their children’s emotions and to work on their emotional understanding and expression. It is a transdiagnostic approach that addresses a broad range of mental health difficulties in children by focusing on helping parents deal with their own as well as their children’s emotions, repairing relationship ruptures, and setting sound boundaries for their children. EFST utilizes both experiential and psychoeducational techniques. In the experiential condition (EXP) emotionally evocative techniques are used such as imaginative two-chair dialogue, evocative empathy, and interventions focused on connecting with emotions. These techniques are intended to help parents process unresolved feelings and deepen their understanding of their child’s emotional reactions. Conversely, the psychoeducational condition (PE) involves the didactic teaching of emotion skills without the use of experiential tasks, providing parents with a rationale for validating emotions and concrete guidance on dealing with difficult situations. The study targets mental health problems expressed through internalizing (INT) and externalizing (EXT) symptoms in children and adolescents. Internalizing symptoms typically include anxiety, depression, and withdrawal, whereas externalizing symptoms are behaviors like aggression and rule breaking. Both the experiential and psychoeducational versions of EFST reduced externalizing behaviors, such as aggression and disruptiveness, as well as internalizing symptoms like anxiety and depression, reported by parents. However, the study’s reliance on parental reports as the primary measure of children’s symptom changes may have introduced bias. Parents’ perceptions may be subjective and affected by their emotional state and expectations. Future research could benefit from incorporating objective measures or reports from multiple sources, such as teachers or the children themselves, to triangulate the findings. Additionally, the long-term sustainability of the intervention’s effects remains unclear. Longitudinal studies are needed to determine whether the benefits of EFST persist over time.

The second study, by Diamond et al. (2016), compares the empirically based experiential therapies Attachment-Based Family Therapy (ABFT) and Emotion-Focused Therapy (EFT). ABFT is a family-based intervention designed to tackle issues within the family system, particularly focusing on unresolved anger in young adults toward their parents. The ABFT model, rooted in structural family therapy and attachment theory, aims to facilitate productive emotional processing to transform working models of self and others, leading to more secure attachment. It has proven effective in clinical trials for depressed and suicidal adolescents. Similarly, EFT integrates client-centered principles with Gestalt techniques to evoke core maladaptive emotions and transform them, thereby adjusting personal narratives and relational action tendencies. The emphasis in both therapies on therapist empathy, validation, and safety is a significant strength, highlighting the importance of the therapeutic alliance in facilitating emotional healing. The focus on attachment and emotional processing is crucial, as unresolved anger can significantly impact relationships and overall mental health.

Although ABFT and EFT differ in their theoretical roots (ABFT emerging from attachment and structural family therapy, and EFT from humanistic and emotion-focused traditions), the study by Diamond et al. (2016) is valuable because it illustrates how two distinct experiential approaches can target similar emotional processes. ABFT uses direct, in-vivo interaction with caregivers to repair relational ruptures, whereas EFT uses chair-based dialogical methods to help clients access and transform core emotional experiences. Examining these approaches side by side helps clarify the range of experiential techniques relevant to resolving unresolved anger and attachment-related distress.

### Group-based interventions

A distinct subset of the included studies implemented experiential interventions in a group format, offering a qualitatively different therapeutic context compared with individual chair-based work. Group-based experiential therapies introduce additional interpersonal and social processes, such as witnessing, emotional resonance, collective validation, and shared meaning-making (Mahon and Leszcz, 2017). These group dynamics can amplify emotional engagement, while also affecting how chair-based tasks are facilitated and experienced (Pascual-Leone et al., 2011).

Hagl et al. (2014) investigated the efficacy of Dialogical Exposure Therapy (DET) compared to a supportive group intervention for women who had lost their husbands. DET utilizes the Gestalt empty-chair method, encouraging participants to engage in an imagined dialogue with their deceased or missing husband to process unresolved emotions and achieve closure. Hagl et al. (2014) found that short-term dialogical exposure therapy was moderately effective in treating traumatically bereaved women, showing some advantages over supportive group therapy.

Arimitsu (2016) investigated the Enhancing Self-Compassion Program (ESCP), which was implemented in group format with four to six participants per group. This intervention was designed to improve self-compassion and related psychological outcomes among Japanese individuals with low self-compassion, highlighting the group dynamic as a key component of the program. The Enhancing Self-Compassion Program (ESCP) is a structured intervention designed to increase self-compassion among individuals, particularly those who are highly self-critical. The program is based on the principles of compassion-focused therapy and incorporates various techniques aimed at fostering self-kindness, common humanity, and mindfulness. However, the study’s sample size (4–6 participants per group) is relatively small, which may have limited the statistical power and the ability to detect significant effects. Additionally, cultural factors could have affected the program’s effectiveness, as self-compassion can be perceived differently depending on the culture. The study’s focus on Japanese individuals highlights the need for cross-cultural research to examine the adaptability of ESP in diverse cultural contexts.

These studies demonstrate that group-based experiential therapies operate through mechanisms that differ meaningfully from those in individual therapy. In Hagl et al. (2014), the Dialogical Exposure Therapy was delivered in a group format, but the Empty-Chair dialogue itself was conducted by one individual at a time. While a participant engaged in the imagined dialogue with the deceased or missing husband, the remaining group members observed and participated in supportive and psychoeducational components of the session. The design comparison (Empty-Chair vs. supportive group discussing their loss) indicates that the differential effect is attributable to the dialogical exposure; observing a peer confront “unfinished business” may also have functioned as a vicarious corrective emotional experience, consistent with known group therapy mechanisms.

Although the Enhancing Self-Compassion Program (ESCP; Arimitsu, 2016) used a small-group format (four to six participants), it included a Three-Chair technique in one session. This exercise involved the Critic, the Criticized, and the Compassionate Self, where the participant shifted between chairs and finally adopted the Compassionate Self perspective. The technique was performed individually with therapist guidance, while other participants either observed the process or contributed during the subsequent group reflection. In this context, observation served a different mechanism than in trauma-focused Chairwork: witnessing others struggle with self-criticism and compassion fostered Common Humanity, normalization, and validation- key mechanisms in self-compassion interventions.

In both studies, the Chairwork component was performed by a single active participant, with other group members primarily in an observer or reflective role. This structure suggests that the therapeutic impact of Chairwork in group settings may extend beyond the active participant through vicarious learning, emotional processing, and group-based validation mechanisms. The broader literature on group-based experiential work highlights the emergence of Emotion-Focused Group Therapy (EFGT) as a distinct therapeutic format. EFGT adapts the principles and tasks of individual Emotion-Focused Therapy to a group context, using both direct emotional work (e.g., chair dialogues performed by individual members) and vicarious emotional processing, in which group members benefit from witnessing others engage in experiential tasks (Thompson and Girz, 2019). A recent review (Song et al., 2024) EFGT research underscores the need for further high-quality research to clarify how experiential techniques operate within group-based settings and to identify the active components that contribute to therapeutic change.

### Couple therapy

Our systematic review found no empirical investigation that specifically measured the use of Chairwork in couple therapy. However, we did identify a book chapter describing the use of Chairwork as a key technique within Schema Therapy for Couples (ST-C; Atkinson and Perris, 2020). The chapter highlights the role of Chairwork in helping couples break negative behavioral patterns and address maladaptive schemas, focused on meeting each partner’s basic emotional needs. Chairwork is depicted as a central intervention in ST-C, employed alongside approaches such as connecting dialogues and rescripting imagery, particularly when addressing challenging couple dynamics. While this provides valuable theoretical insights into the use of Chairwork within ST-C, the lack of empirical studies on its specific efficacy in couple therapy settings indicates a gap that should be addressed in future research.

The article by Beckley (2022) offers further context on how Schema Theory can help explain interpersonal dynamics in relationships with intimate partner violence (IPV). The article illustrates how early relational patterns can resurface within intimate relationships, potentially escalating into harmful behaviors. It suggests that misunderstandings of the perpetrator’s motivations, particularly those rooted in power and control, can sometimes hinder deeper exploration of underlying relational trauma. This framework does not blame the victim and emphasizes that a deeper understanding of the partners’ trauma history may help elucidate the triggers of violence or coercive control. This perspective further supports the notion that Schema Therapy, including interventions like Chairwork, could be valuable in addressing complex relational dynamics; however, there is limited empirical research on its application in these contexts.

## Limitations and future research

In our systematic review, we focused solely on randomized controlled trial designs given their status as the gold standard for testing intervention efficacy (Shadish et al., 2002). However, despite rigorous designs, not all the selected RCTs were consistent in reporting effect sizes. This limited our ability to directly compare the magnitude of treatment effects across studies. It would be beneficial if future studies examining Chairwork-based interventions systematically reported effect sizes for all the assessed outcomes.

In many studies, low sample sizes were identified as a significant limitation, affecting the generalizability of their results. Smaller sample sizes reduce the statistical power and increase the margin of error, making it difficult to draw robust conclusions that apply to broader populations. The demographic characteristics of the samples studied present a significant limitation. The included RCTs often studied samples with highly heterogeneous or insufficiently reported demographics (e.g., age, gender distribution, cultural background). This lack of detail and the narrow focus of some samples affect the external validity and generalizability of the findings to diverse clinical populations. A further limitation is that all the studies relied solely on self-report instruments to assess outcomes. This reliance introduces potential biases, as self-report measures can be influenced by various subjective factors, which affects the accuracy and reliability of the findings.

Additionally, the studies employed a diverse range of Chairwork interventions for a variety of problems. The heterogeneity of the interventions and lack of clarity regarding the specific components used further complicate the synthesis of findings. This variability makes it hard to determine precisely which aspects of the interventions are differentially effective. It is therefore important to conduct both efficacy research and process research.

The limitations of the review itself need to be more carefully considered. We focused only on studies with a quantitative RCT design and some form of control group (waiting list, compared to other interventions, etc.). That means that some published case studies or analog studies verifying the effectiveness of Chairwork were not included in our review. Crucially, the substantial heterogeneity of the interventions and the inconsistent reporting of effect sizes across the included RCTs precluded the feasibility of conducting a quantitative meta-analysis. Therefore, this systematic review was limited to a narrative synthesis of the findings.

Another limitation of our systematic review was the restriction to English language studies. We sought to minimize the risk of missing studies that questioned the efficacy of Chairwork by including PhD theses. Interestingly, all the studies identified supported the efficacy of Chairwork. A third limitation of our systematic review is that outcomes of the same type cannot be compared due to the lack of reported findings in primary studies and the selective assessment of treatment effects.

## Data Availability

The original contributions presented in the study are included in the article/Supplementary material, further inquiries can be directed to the corresponding author.
