# Supplementary material for: Experiential therapies including Chairwork: a systematic review of randomized controlled trials
Source: Front Psychol. 2026 Jan 23;16:1692630. doi: 10.3389/fpsyg.2025.1692630 (PMC12876151; doi:10.3389/fpsyg.2025.1692630)
Supplement: Supplementary file 1 [file Data_Sheet_1.docx]

**This document contains supplementary information for the manuscript “Experiential Therapies Including Chairwork: A Systematic Review of Randomized Controlled Trials”. It provides detailed methodological data and additional results that support the findings presented in the main text.**

It includes:

Appendix A: Search Syntax used for the systematic literature search.

Appendix B: List of eligible studies meeting the exclusion criteria.

Appendix C: Table detailing the measured constructs and scales used in the included studies.

**AppendixA
Search Syntax**

“chairwork” OR “chair-work” OR “empty chair” OR “empty-chair” OR “two chair” OR “two-chair” OR “unfinished business” OR "hot seat" OR "imaginal dialogue" OR "imaginal confrontation" OR "self-critical chair" OR "worry dialogue" OR "self-interruptive chair" OR "self-soothing chair" OR "self-interruptive chair" OR "compassionate chair" OR "multiple selves" OR "multi self" OR "multi-self" OR "multi mind" OR "multiplicity of self" OR "second-person dialogue" OR "second person dialogue" OR "voice dialogue" OR "voice therapy"

AND “psychotherapy” OR “therapy” OR “counselling” OR “counseling” OR” intervention”

AND “outcome” OR “efficacy” OR "effect" OR "change" OR “effectiveness”

**Appendix B**

**Eligible Studies Meeting the Exclusion Criteria (n=33)**

Avsar, V., & Sevim, S. A. (2022). The Effectiveness of Cognitive Behavioral Therapy Including Updating the Early Life Experiences and Images with the Empty Chair Technique on Social Anxiety. International Journal of Assessment Tools in Education, 181–202. https://doi.org/10.21449/ijate.1062613

Bell, T., Montague, J., Elander, J., & Gilbert, P. (2020). “A definite feel‐it moment”: Embodiment, externalisation and emotion during chair‐work in compassion‐focused therapy. Counselling and Psychotherapy Research, 20(1), 143–153. https://doi.org/10.1002/capr.12248

Beuchat, H., Grandjean, L., Despland, J., Pascual‐Leone, A., Gholam, M., Swendsen, J., & Kramer, U. (2022). Ecological momentary assessment of emotional processing: An exploratory analysis comparing daily life and a psychotherapy analogue session. Counselling and Psychotherapy Research, 22(2), 345–356. https://doi.org/10.1002/capr.12455

Conoley, (1983). the effect of the abcs of rational emotive therapy and the empty-chair technique of gestalt-therapy on anger reduction. psychotherapy-theory research and practice.

de Oliveira, I. R., Hemmany, C., Powell, V. B., Bonfim, T. D., Duran, É. P., Novais, N., Velasquez, M., Di Sarno, E., Alves, G. L., & Cesnik, J. A. (2012). Trial-based psychotherapy and the efficacy of trial-based thought record in changing unhelpful core beliefs and reducing self-criticism. CNS Spectrums, 17(1), 16–23. https://doi.org/10.1017/S1092852912000399

Delavechia, T. R., Velasquez, M. L., Duran, É. P., Matsumoto, L. S., & Oliveira, I. R. de. (2016). Changing negative core beliefs with trial-based thought record. Archives of Clinical Psychiatry (São Paulo), 43(2), 31–33. https://doi.org/10.1590/0101-60830000000078

Diamond, G. M., Rochman, D., & Amir, O. (2010). Arousing primary vulnerable emotions in the context of unresolved anger: “Speaking about” versus “speaking to”. Journal of Counseling Psychology, 57(4), 402–410. https://doi.org/10.1037/a0021115

Falconer, C. J., Slater, M., Rovira, A., King, J. A., Gilbert, P., Antley, A., & Brewin, C. R. (2014). Embodying Compassion: A Virtual Reality Paradigm for Overcoming Excessive Self-Criticism. PLoS ONE, 9(11), e111933. https://doi.org/10.1371/journal.pone.0111933

Field, N. P., & Bonanno, G. A. (2001). The Role of Blame in Adaptation in the First 5 Years Following the Death of a Spouse. American Behavioral Scientist, 44(5), 764–781. https://doi.org/10.1177/00027640121956485

Field, N. P., & Horowitz, M. J. (1998). Applying an Empty-Chair Monologue Paradigm to Examine Unresolved Grief. Psychiatry, 61(4), 279–287. https://doi.org/10.1080/00332747.1998.11024840

Field, N. P., Bonanno, G. A., Williams, P., & Horowitz, M. J. (2000). Appraisals of blame in adjustment in conjugal bereavement. Cognitive Therapy and Research, 24(5), 551–569. https://doi.org/10.1023/A:1005514128798

Greenberg, L. S., & Clarke, K. M. (1979). Differential effects of the two-chair experiment and empathic reflections at a conflict marker. Journal of Counseling Psychology, 26(1), 1–8. https://doi.org/10.1037/0022-0167.26.1.1

Greenberg, L. S., & Dompierre, L. M. (1981). Specific effects of Gestalt two-chair dialogue on intrapsychic conflict in counseling. Journal of Counseling Psychology, 28(4), 288–294. https://doi.org/10.1037/0022-0167.28.4.288

Greenberg, L. S., & Higgins, H. M. (1980). Effects of two-chair dialogue and focusing on conflict resolution. Journal of Counseling Psychology, 27(3), 221–224. <https://doi.org/10.1037/0022-0167.27.3.221>

Greenberg, L. S., & Malcolm, W. (2002). Resolving unfinished business: Relating process to outcome. Journal of Consulting and Clinical Psychology, 70(2), 406–416. <https://doi.org/10.1037/0022-006X.70.2.416>

Greenberg, L. S., & Webster, M. C. (1982). Resolving decisional conflict by Gestalt two-chair dialogue: Relating process to outcome. Journal of Counseling Psychology, 29(5), 468–477. https://doi.org/10.1037/0022-0167.29.5.468

Kramer, U., Beuchat, H., Grandjean, L., Despland, J.-N., & Pascual-Leone, A. (2023). Change in emotional processing in daily life: relationship with in-session self-esteem. Counselling Psychology Quarterly, 36(2), 235–250. https://doi.org/10.1080/09515070.2022.2029349

Lafrance Robinson, A., McCague, E. A., & Whissell, C. (2014). “That chair work thing was great”: a pilot study of group-based emotion-focused therapy for anxiety and depression. Person-Centered & Experiential Psychotherapies, 13(4), 263–277. https://doi.org/10.1080/14779757.2014.910131

Maslove. (1989). The differential effects of empathic reflection and the Gestalt emptychair dialogue on depth of experiencing when used with an issue of unfinished business.

McKinnon, J. M., & Greenberg, L. S. (2017). Vulnerable Emotional Expression In Emotion Focused Couples Therapy: Relating Interactional Processes To Outcome. Journal of Marital and Family Therapy, 43(2), 198–212. https://doi.org/10.1111/jmft.12229

Nardone, S., Pascual-Leone, A., & Kramer, U. (2022). “Strike while the iron is hot”: Increased arousal anticipates unmet needs. *Counselling Psychology Quarterly*, *35*(1), 110–128. https://doi.org/10.1080/09515070.2021.1955659

Narkiss-Guez, T., Enav Zichor, Y., Guez, J., & Diamond, G. M. (2015). Intensifying attachment-related sadness and decreasing anger intensity among individuals suffering from unresolved anger: The role of relational reframe followed by empty-chair interventions. Counselling Psychology Quarterly, 28(1), 44–56. https://doi.org/10.1080/09515070.2014.924480

Reidar Stiegler, J., Uleberg Vildalen, V., Heggem, T., Båfjord Ismaili, S., & Schanche, E. (2023). The effect of the <scp>two‐chair</scp> dialogue intervention on <scp>self‐compassion</scp> ‐ adding an emotional evocative component to a basic Rogerian condition. Counselling and Psychotherapy Research, 23(2), 349–358. https://doi.org/10.1002/capr.12534

Rochman, D., & Diamond, G. M. (2008). From unresolved anger to sadness: Identifying physiological correlates. *Journal of Counseling Psychology*, *55*(1), 96–105. https://doi.org/10.1037/0022-0167.55.1.96

Shahar, B., Bar-Kalifa, E., & Alon, E. (2017). Emotion-focused therapy for social anxiety disorder: Results from a multiple-baseline study. Journal of Consulting and Clinical Psychology, 85(3), 238–249. https://doi.org/10.1037/ccp0000166

Shahar, B., Carlin, E. R., Engle, D. E., Hegde, J., Szepsenwol, O., & Arkowitz, H. (2012). A Pilot Investigation of Emotion‐Focused Two‐Chair Dialogue Intervention for Self‐Criticism. Clinical Psychology & Psychotherapy, 19(6), 496–507. https://doi.org/10.1002/cpp.762

Stiegler, J. R., Molde, H., & Schanche, E. (2018a). Does an emotion‐focused two‐chair dialogue add to the therapeutic effect of the empathic attunement to affect? Clinical Psychology & Psychotherapy, 25(1). https://doi.org/10.1002/cpp.2144

Stiegler, J. R., Molde, H., & Schanche, E. (2018b). Does the two-chair dialogue intervention facilitate processing of emotions more efficiently than basic Rogerian conditions? European Journal of Psychotherapy & Counselling, 20(3), 337–355. https://doi.org/10.1080/13642537.2018.1495245

Thompson, S., & Girz, L. (2020). Overcoming shame and aloneness: Emotion-focused group therapy for self-criticism. Person-Centered & Experiential Psychotherapies, 19(1), 1–11. https://doi.org/10.1080/14779757.2019.1618370

Toker ugurlu, tugce, kalkan oguzhanoglu, nalan, & atesci, figen. (2020). Effect of Psychodrama Group Therapy on Remission and Relapse in Opioid Dependence. Archives of Neuropsychiatry. https://doi.org/10.29399/npa.25001

van Maarschalkerweerd, F. A. T., Engelmoer, I. M., Simon, S., & Arntz, A. (2021). Addressing the punitive parent mode in schema therapy for borderline personality disorder: Short-term effects of the empty chair technique as compared to cognitive challenging. Journal of Behavior Therapy and Experimental Psychiatry, 73, 101678. https://doi.org/10.1016/j.jbtep.2021.101678

Vrana, G. C. (2010). Changing emotion with emotion versus changing emotion with cognition: A therapy-analogue study. Dissertation/Thesis.

Whelton, W. J., & Greenberg, L. S. (2005). Emotion in self-criticism. Personality and Individual Differences, 38(7), 1583–1595. <https://doi.org/10.1016/j.paid.2004.09.024>

**Appendix C**

**Table detailing the measured constructs and scales used in the included studies.**

| **Reference** | **Measured construct/ scales** |
| --- | --- |
| Ansar et al., 2022 | Brief Problem Monitor for teachers (BPM-T; Achenbach & Rescorla, 2001)  Brief Problem Monitor for parents (BPM-T; Achenbach & Rescorla, 2001)  Working Alliance Inventory (WAI; Horvath & Greenberg, 1989) |
| Arimitsu (2016) | Self-Compassion Scale (SCS; Neff, 2003)  Self-esteem Scale Scale (RSS; Rosenberg, 1965)  Beck Depression Inventory-II (BDI-II; Beck et al., 1996)  State-Trait Anxiety Inventory (STAI-T; Spielberger et al., 1983)  Depression Anxiety Cognition Scale (DACS; Fukui, 1998)  Multiple Mood Scale (MMS; Terasaki et al., 1992)  Self-Conscious Emotion Scale (SCES; Arimitsu, 2005) |
| Clarke  (1981) | Scale of Vocational Indecision (SVI; Osipow and Carney, 1975)  Assessment of Career Decision-Making (ACDM; Harren, 1975) |
| Diamond et al., (2016) | Unfinished Business Resolution Scale (UFB-RS; Singh, 1994)  State-Trait Anger Expression Inventory (STAXI; Spielberger, 1996)  Brief Symptom Inventory (BSI; Derogatis & Melisaratos, 1983)  Experiences in Close Relationships – Relationship Structures Questionnaire  (ECR-RS; Fraley et al., 2011)  Client Emotional Productivity Scale-Revised (CEPS-R; Greenberg et al., 2007) |
| Duran et al., (2020) | Beck Depression Inventory-II; (BDI-II;Beck et al., 1996)  Davidson Trauma Scale (DTS; Davidson et al., 1997)  Beck Anxiety Inventory (BAI; Beck et al., 1988)  Dysfunctional Attitude Scale (DAS; Weissman & Beck, 1978) |
| Ellison et al., (2009) | Beck Depression Inventory (BDI; Beck et al. 1961)  Symptom Checklist–90—Revised (SCL-90–R; Derogatis, 1983)  Inventory of Interpersonal Problems (IIP; Horowitz et al., 1988)  Rosenberg Self-Esteem Scale (RSE; Rosenberg, 1965)  Longitudinal Interval Follow-up Evaluation-II (LIFE-II; Keller et al., 1987) |
| Glisenti et al. (2021) | Credibility/Expectancy Questionnaire  (CEQ; Devilly & Borkovec, 2000)  Eating Disorder Examination Questionnaire  (EDE-Q-6.0; Fairburn & Beglin, 1994)  Binge Eating Scale (BES; Gormally et al., 1982) |
| Goldman et al., (2006) | Beck Depression Inventory (BDI; Beck et al. 1961)  Symptom Checklist-90-Revised (SCL-90-R; Derogatis, 1977)  Inventory of Interpersonal Problems (IIP; Horowitz et al., 1988)  Rosenberg Self-Esteem Scale (RSE; Rosenberg, 1965)  Global Severity Index (GSI; Derogatis, 1993)  Barrett-Lennard Relationship Inventory (BLRI; Barrett-Lennard, 1962)  Truax Accurate Empathy Scale (Truax, 1967)  Task-Specific Intervention Adherence  (Greenberg & Watson, 1998). |
| Greenberg et al., (2008) | The Enright Forgiveness Inventory (EFI; Enright et al., 2000)  Unfinished Business Empathy and Acceptance Scale (UFB EA; Singh, 1994)  Unfinished Business Feelings and Needs Scale (UFBFN; Singh, 1994)  Target Complaints (TC) Discomfort and Change Scale (Battle et al., 1968)  Global Symptom Index (GSI) of the Symptom Checklist-90—Revised  (SCL-90 –R; Derogatis, 1983)  Beck Depression Inventory (BDI; Beck et al., 1961).  Emotional Arousal Session Report Measure (Warwar & Greenberg, 2002)  Working Alliance Inventory (WAI; Horvath & Greenberg, 1989) |
| Greenberg & Watson (1998) | Beck Depression Inventory (BDI; Beck et al., 1961)  Symptom Checklist-9O-Revised (SCL-90-R; Derogatis et al., 1976)  Rosenberg Self-Esteem Scale (RSES; Rosenberg, 1989)  Inventory of Interpersonal Problems (IIP; Horowitz et al., 1988)  Target Complaints (TCBS; Battle et al, 1966).  Longitudinal Interval Follow-Up Evaluation ZZ (LIFE; Keller et al, 1987)  Working Alliance Inventory (WAI; Horvath & Greenberg, 1989)  Barrett-Lennard Perceived Empathy (BLRI; Barrett-Lennard, 1962)  Truax Accurate Empathy Scale (Truax, 1967) |
| Hagl et al., (2014) | Posttraumatic Diagnostic Scale (PDS; Foa et al., 1997)  General Health Questionnaire (GHQ-28; Goldberg & Hillier, 1979)  Impact of Event Scale (IES; Horowitz et al., 1979)  Grief Inventory (GI; Layne et al., 2001) |
| Chagigiorgis (2009) | Childhood Trauma Questionnaire (CTQ; Bernstein & Fink, 1993)  Personality Diagnostic Questionnaire–Fourth Edition (PDQ-4; Hyler, 1994).  PTSD Symptom Severity Interview (PSSI; Foa et al., 1993).  Symptom Checklist-Revised (SCL-90-R; Derogatis, 1983).  Beck Depression Inventory-II (BDI-II; Beck et al., 1996).  State-Trait Anxiety Inventory (STAI; Speilberger et al., 1970).  Impact of Event Scale (IES; Horowitz, 1986)  Inventory of Interpersonal Problems (IIP; Horowitz et al., 1988).  Rosenberg Self Esteem Scale (RSES; Rosenberg, 1989)  Resolution Scale (RS; Singh, 1994)  Levels of Engagement Scale (LES; Paivio et al., 2001)  Post Session Questionnaire (PSQ; Paivio et al., in press).  Working Alliance Inventory (WAI; Horvath & Greenberg, 1989) |
| de Oliveira et al., (2012) | Liebowitz Social Anxiety Scale (LSAS; Liebowitz, 1987)  Fear of Negative Evaluation Scale (FNE; Watson & Friend, 1969)  Social Avoidance and Distress Scale (SADS; Watson & Friend, 1969)  Beck Anxiety Inventory (BAI; Beck et al., 1988)  Clinical Global Impression – Improvement (CGI-I; Guy, 1976) |
| Paivio et al., (2010) | Impact of Event Scale (IES; Horowitz,1986)  State Trait Anxiety Inventory (Spielberger et al., 1970)  Beck Depression Inventory-II (BDI-II; Beck et al., 1996)  Target Complaints (Discomfort) Scale (TCD; Battle et al., 1966)  Rosenberg Self-Esteem Scale (RSE; Rosenberg, 1989)  Inventory of Interpersonal Problems (IPP; Horowitz et al., 1988)  Resolution Scale (RS; Singh, 1994)  Pretreatment Predictor Measures:  Childhood Trauma Questionnaire (CTQ; Bernstein & Fink, 1998)  Symptom Severity Interview (PSSI; Foa et al., 1993)  Personality Diagnostic Questionnaire (PDQ-4; Hyler, 1994)  Process Measures:  Working Alliance Inventory (WAI; Horvath &Greenberg, 1989)  Adherence Checklist (ADH; Paivio & Nieuwenhuis, 2001)  Therapist Facilitating Scale (TFS; Hall, 2007)  Subjective Units of Distress (SUDS) |
| Paivio & Greenberg (1995) | Symptom Checklist-90-Revised (SCL-90-R; Derogatis, 1977)  Inventory of Interpersonal Problems (IIP; Horowitz et al., 1988)  Target Complaints (TC; Battle et al., 1966)  Unfinished Business Resolution Scale; (UFB-RS; Singh, 1994)  Structural Analysis of Social Behavior; (SASB; Benjamin, 1988)  Working Alliance Inventory (WAI; Horvath & Greenberg, 1989) |
| Parker (2007) | Anger Parameters Scale (APS; Fernandez, 2001)  Anger Expressions Scale (AES; Fernandez, 2001)  Anger diaries |
| Ralston (2006) | Client Characteristic Measures:  Childhood Trauma Questionnaire (CTQ; Bernstein et al., 1994)  Symptom Severity Interview (PSSI; Foa et al., 1993)  Toronto Alexithymia Scale (TAS-20; Bagby et al., 1994)  Personality Diagnostic Questionnaire 4+ (PDQ-4+; Hyler, 1994)  Outcome Measures:  The Symptom Checklist-90-Revised  (SCL-90-R; Derogatis, 1983)  Inventory of Interpersonal Problems (IIP; Horowitz et al., 1988)  Resolution Scale (RS; Singh, 1994)  Rosenberg Self-Esteem Scale (RSE; Rosenberg, 1965)  Impact of Events Scale (IES; Horowitz, 1986)  Process Measures:  The Working Alliance Inventory (WAI; Horvath & Greenberg, 1989)  Patient Experiencing (EXP) Scale (Klein et al., 1969; Klein et al., 1986)  Client’s Emotional Arousal Scale-Revised (EAS-R; Machado et al., 1999)  Postsession Questionnaire (PSQ) |
| Rodrigues et al., (2023) | Yale-Brown Obsessive Compulsive Scale (Y-BOCS; Goodman et al., 1989)  Beck Anxiety Inventory (BAI; Beck et al., 1988)  Beck Depression Inventory (BDI; Beck et al., 1996) |
| Souliere (1995) | Symptom Checklist-90-Revised (SCL-90-R; Derogatis, 1977)  The Working Alliance Inventory (WAI; Horvath & Greenberg, 1989)  Unfinished Business Resolution Scale; (UFB-RS; Singh, 1994)  Structural Analysis of Social Behavior; (SASB; Benjamin, 1988)  Target Complaint Discomfort Box Scale (TCDBS)  State-Trait Anger Expression Inventory (STAXI; Spielberger, 1996) |
| Stefan et al., 2023 | Liebowitz Social Anxiety Scale – Self-Report (LSAS-SR; Liebowitz, 1987)  Brief Fear of Negative Evaluation Scale-Revised (BFNE-II; Leary, 1983)  Acceptance and Action Questionnaire – II (AAQ-2; Bond et al., 2011) |
| Trachsel et al., (2012) | Ambivalence regarding the continuation or separation of the relationship  (ARCOS; Trachsel & Boller, 2008)  Perceived Stress Questionnaire (PSQ; Levenstein et al., 1993)  Center for epidemiologic studies depression scale (CES-; Radloff, 1977)  Satisfaction with life scale (SWLS; Diener et al., 1985)  brief symptom inventory (BSI (Derogatis, 1993)  Lübeck alcohol dependence and abuse screening test (LAST; Rumpf, et al., 2001)  Drug abuse screening test (DAST-10; Cocco & Carey, 1998)  Bern post-session report 2000 (BPSR-T/P; Flückiger et al., 2010) |
| Watson et al. (2003) | Beck Depression Inventory (BDI; Beck et al., 1961)  Inventory of Interpersonal Problems (IIP; Horowitz et al., 1988)  Rosenberg Self-Esteem Scale (RSE; Rosenberg, 1965)  Symptom Checklist-90—Revised (SCL-90 –R; Derogatis et al., 1976).  Dysfunctional Attitude Scale (DAS; Weissman & Beck, 1978)  Problem-Focused Style of Coping (PF-SOC; Heppner et al., 1995) |

**References**

Achenbach, T. M., & Rescorla, L. A. (2001). Manual for the ASEBA school-age forms and profiles. Research Center for Children, Youth, and Families, University of Vermont.

Achenbach, T. M., McConaughy, S. H., Ivanova, M. Y., & Rescorla, L. A. (2011). Manual for the ASEBA brief problem monitor (BPM). University of Vermont.

Arimitsu, K. (2005). Zaiakukan, haji to seishin-tekikenkou nintinoyugami no kankei [Exploring the relationships among guilt, shame, embarrassment, cognitive distortions, and mental health]. Poster presented at the 5th annual meeting of the Japan Association for Cognitive Therapy, Tokyo, Japan.

Bagby, R. M., Parker, J. D. A., & Taylor, G. J. (1994). The twenty-item Toronto Alexithymia Scale—I. Item selection and cross-validation of the factor structure. Journal of Psychosomatic Research, 38(1), 23–32. https://doi.org/10.1016/0022-3999(93)90120-5

Barrett-Lennard, G. T. (1962). Dimensions of therapist response as causal factors in therapeutic change. Psychological Monographs: General and Applied, 76(43), 1–36. https://doi.org/10.1037/h0093918

Battle, C. C., Imber, S. D., Hoehn-Saric, R., Stone, A. R., Nash, E. R., & Frank, J. D. (1966). Target complaints as criteria of improvement. American Journal of Psychotherapy, 20(1), 184–192.

Beck, A. T., Epstein, N., Brown, G., & Steer, R. A. (1988). An inventory for measuring clinical anxiety: Psychometric properties. Journal of Consulting and Clinical Psychology, 56(6), 893–897. https://doi.org/10.1037/0022-006X.56.6.893

Bernstein, D. P., & Fink, L. (1993). Childhood Trauma Questionnaire: A retrospective self-report manual. Psychological Corporation.

Cocco, K. M., & Carey, K. B. (1998). Psychometric properties of the Drug Abuse Screening Test in psychiatric outpatients. Psychological Assessment, 10(4), 408–414. https://doi.org/10.1037/1040-3590.10.4.408

Davidson, J. R., Book, S. W., Colket, J. T., Tupler, L. A., Roth, S., David, D., Hertzberg, M., Mellman, T., Beckham, J. C., & Smith, R. D. (1997). Assessment of a new self-rating scale for post-traumatic stress disorder. Psychological Medicine, 27(1), 153–160. https://doi.org/10.1017/S0033291796004751

Derogatis, L. R. (1977). SCL-90-R: Administration, scoring, and procedures manual-I for the revised version. Johns Hopkins University School of Medicine.

Derogatis, L. R. (1983). SCL-90-R: Symptom Checklist-90-R. Administration, scoring, and procedures manual. National Computer Systems.

Derogatis, L. R. (1993). BSI: Brief Symptom Inventory: Administration, scoring, and procedures manual (4th ed.). National Computer Systems.

Devilly, G. J., & Borkovec, T. D. (2000). Psychometric properties of the credibility/expectancy questionnaire. Journal of Behavior Therapy and Experimental Psychiatry, 31(2), 73–86. https://doi.org/10.1016/S0005-7916(00)00012-4

Diener, E., Emmons, R. A., Larsen, R. J., & Griffin, S. (1985). The Satisfaction with Life Scale. Journal of Personality Assessment, 49(1), 71–75. https://doi.org/10.1207/s15327752jpa4901_13

Fairburn, C. G., & Beglin, S. J. (1994). Assessment of eating disorders: Interview or self-report questionnaire? International Journal of Eating Disorders, 16(4), 363–370. <https://doi.org/10.1002/1098-108X(199412)16:4>

Flückiger, C., Regli, D., Zwahlen, D., Hostettler, S., & Caspar, F. (2010). Der Berner Patienten- und Therapeutenstundenbogen 2000. Ein Instrument zur Erfassung von Therapieprozessen [The Bern Post Session Report 2000 patient and therapist versions: Measuring psychotherapeutic processes]. Zeitschrift für Klinische Psychologie und Psychotherapie, 39(2), 71–79. https://doi.org/10.1026/1616-3443.39.2.71

Foa, E. B., Riggs, D. S., Dancu, C. V., & Rothbaum, B. O. (1993). Reliability and validity of a brief instrument for assessing post-traumatic stress disorder. Journal of Traumatic Stress, 6(4), 459–473.

Fraley, R. C., Waller, N. G., & Brennan, K. A. (2000). An item response theory analysis of self-report measures of adult attachment. Journal of Personality and Social Psychology, 78(2), 350–365.

Fukui, I. (1998). Development of depression and anxiety cognition scale: Toward the construction of a cognitive-behavioral model of depression and anxiety. Japanese Journal of Behavior Therapy, 24, 57–70.

Goldberg, D. P., & Hillier, V. F. (1979). A scaled version of the General Health Questionnaire. Psychological Medicine, 9(1), 139–145. <https://doi.org/10.1017/S0033291700021644>

Goodman, W. K., Price, L. H., Rasmussen, S. A., Mazure, C., Fleischmann, R. L., Hill, C. L., Heninger, G. R., & Charney, D. S. (1989). The Yale-Brown Obsessive Compulsive Scale: I. Development, use, and reliability. Archives of General Psychiatry, 46(11), 1006–1011. https://doi.org/10.1001/archpsyc.1989.01810110048007

Gormally, J., Black, S., Daston, S., & Rardin, D. (1982). The assessment of binge eating severity among obese persons. Addictive Behaviors, 7(1), 47–55. <https://doi.org/10.1016/0306-4603(82)90024-7>

Horowitz, M. J. (1986). Stress response syndromes (2nd ed.). Jason Aronson.

Horowitz, M. J., Wilner, N., & Alvarez, W. (1979). Impact of Event Scale: A measure of subjective stress. Psychosomatic Medicine, 41(3), 209–218. https://doi.org/10.1097/00006842-197905000-00004

Horvath, A. O., & Greenberg, L. S. (1989). Development and validation of the Working Alliance Inventory. Journal of Counseling Psychology, 36(2), 223–233. https://doi.org/10.1037/0022-0167.36.2.223

Hyler, S. E. (1994). PDQ-4+: Personality Diagnostic Questionnaire-4 Plus. Unpublished manuscript, New York State Psychiatric Institute.

Keller, M. B., Lavori, P. W., Friedman, B., Nielsen, E., Endicott, J., McDonald-Scott, P., & Andreasen, N. C. (1987). The Longitudinal Interval Follow-up Evaluation: A comprehensive method for assessing outcome in prospective longitudinal studies. Archives of General Psychiatry, 44(6), 540–548.

Levenstein, S., Prantera, C., Varvo, V., Scribano, M. L., Berto, E., Luzi, C., & Andreoli, A. (1993). Development of the Perceived Stress Questionnaire: A new tool for psychosomatic research. Journal of Psychosomatic Research, 37(1), 19–32. https://doi.org/10.1016/0022-3999(93)90120-5

Machado, P. P., Beutler, L. E., & Greenberg, L. S. (1999). Revised emotional arousal scale: A procedure for measuring strategy enactment in experiential therapy. Clinical Psychology & Psychotherapy, 6(2), 146–155.

Neff, K. D. (2003). The development and validation of a scale to measure self-compassion. Self and Identity, 2, 223–250.

Osipow, S. H., & Carney, C. G. (1975). Scale of Vocational Indecision. Measurement and Evaluation in Counseling and Development, 8(3), 153–156.

Radloff, L. S. (1977). The CES-D scale: A self-report depression scale for research in the general population. Applied Psychological Measurement, 1(3), 385–401.

Rosenberg, M. (1965). Society and the adolescent self-image. Princeton University Press.

Singh, M. (1994). Validation of a measure of session outcome in the resolution of unfinished business [Unpublished doctoral dissertation]. York University.

Spielberger, C. D., Gorsuch, R. L., & Lushene, R. E. (1970). STAI manual for the state-trait anxiety inventory. Consulting Psychologists Press.

Terasaki, M., Kishimoto, Y., & Koga, A. (1992). Construction of a multiple mood scale. Japanese Journal of Psychology, 62, 350–356.

Truax, C. B. (1967). A scale for the measurement of accurate empathy. In C. R. Rogers, E. T. Gendlin, D. J. Kiesler, & C. B. Truax (Eds.), The therapeutic relationship and its impact: A study of psychotherapy with schizophrenics (pp. 555–568). University of Wisconsin Press.

Watson, D., & Friend, R. (1969). Measurement of social-evaluative anxiety. Journal of Consulting and Clinical Psychology, 33(4), 448–457. https://doi.org/10.1037/h0027806

Weissman, A. N., & Beck, A. T. (1978, August). Development and validation of the Dysfunctional Attitudes Scale: A preliminary investigation. Paper presented at the Annual Convention of the American Psychological Association, Toronto, Ontario, Canada.
